# Quantifying carbon reductions from mode substitution through shared electric mobility hubs in Greater Manchester

**DOI:** 10.1038/s41598-025-22719-3

**Published:** 2025-11-05

**Authors:** Haoyu Wang, Margaret C. Bell, Jingxin Dong, Matthew Burke, Huseyin Ayan, Dilum Dissanayake

**Affiliations:** 1 Newcastle Business School, Northumbria University, Newcastle upon Tyne, NE1 8ST UK; 2 School of Engineering, Newcastle University, Newcastle upon Tyne, NE1 7RU UK; 3 Business School, Newcastle University, Newcastle upon Tyne, NE1 4SE UK; 4 School of Geography, Earth and Environmental Sciences, University of Birmingham, Birmingham, B15 2TT UK

**Keywords:** Shared electric mobility, eHUBS, Mode substitution, Carbon emissions, Net-zero emissions, Ecology, Environmental sciences, Environmental social sciences

## Abstract

This study investigates the attitudes of 1139 participants towards eHUBS usage and daily travel patterns to assess the effectiveness of shared electric mobility hubs (eHUBS) in Greater Manchester. It explores the potential of eHUBS to replace conventional travel modes, including motor vehicles, public transport, and zero-carbon alternatives such as walking and cycling, while examining their integration with public transport and the associated carbon reduction benefits. Analysis indicates a consistent substitution rate of 30% to 35%, suggesting that eHUBS could increase public transport usage by 8% to 13% and reduce overall carbon emissions by 15% to 18% for journeys exceeding 5 km. The findings reveal that eHUBS, particularly e-bikes and e-cargo bikes, can enhance public transport utilisation for short-distance trips, while electric cars reduce reliance on motor vehicles for longer journeys. Although substituting walking and cycling with eHUBS marginally raises emissions for shorter trips, this impact is minimal compared to the overall reduction achieved. This study underscores the significant role of eHUBS in supporting modal shifts and emissions reduction, contributing to more sustainable urban mobility.

**Supplementary Information:**

The online version contains supplementary material available at 10.1038/s41598-025-22719-3.

## Introduction

In the transport sector, governments globally are intensifying efforts to curb carbon emissions, targeting carbon neutrality by 2050^1^. Private motor vehicles, a major source of urban carbon emissions, pose ongoing challenges in cities worldwide. Reducing reliance on private cars is critical for alleviating congestion and lowering emissions, highlighting the need for a shift towards sustainable transport^2^. Within this context, shared mobility has emerged as a viable sustainable alternative in numerous cities^3^. This includes options such as car-sharing, bike and e-scooter rentals, and private transit, which complement traditional public transport^4^. Furthermore, multi-modal shared electric mobility plays a significant role in reducing travel emissions.

Many global cities have already adopted shared e-mobility. For example, widespread e-bike sharing exists in Kunming^5^ and Shenzhen^6^ in China, Richmond^7^ in the U.S., Stuttgart in Germany, Milan in Italy, Copenhagen in Denmark, and Tricity in Poland^8^. E-scooter services are also widely implemented across cities in Australia^9^, France^10^, and in U.S. cities such as Austin, Kansas City, and Portland^11^. In Switzerland^12^ and in Austria and Germany, e-cargo bike sharing is also growing^13^. E-car sharing initiatives are underway in Sweden^14^, Germany (Berlin)^15^, and the UK^16^. These examples demonstrate the global adoption of shared electric mobility, though typically offered in single modes. Comprehensive research on the integrated use of these modes to assess their collective impact on shifting travel behaviours and supporting low-carbon transport remains limited.

**Fig. 1:**
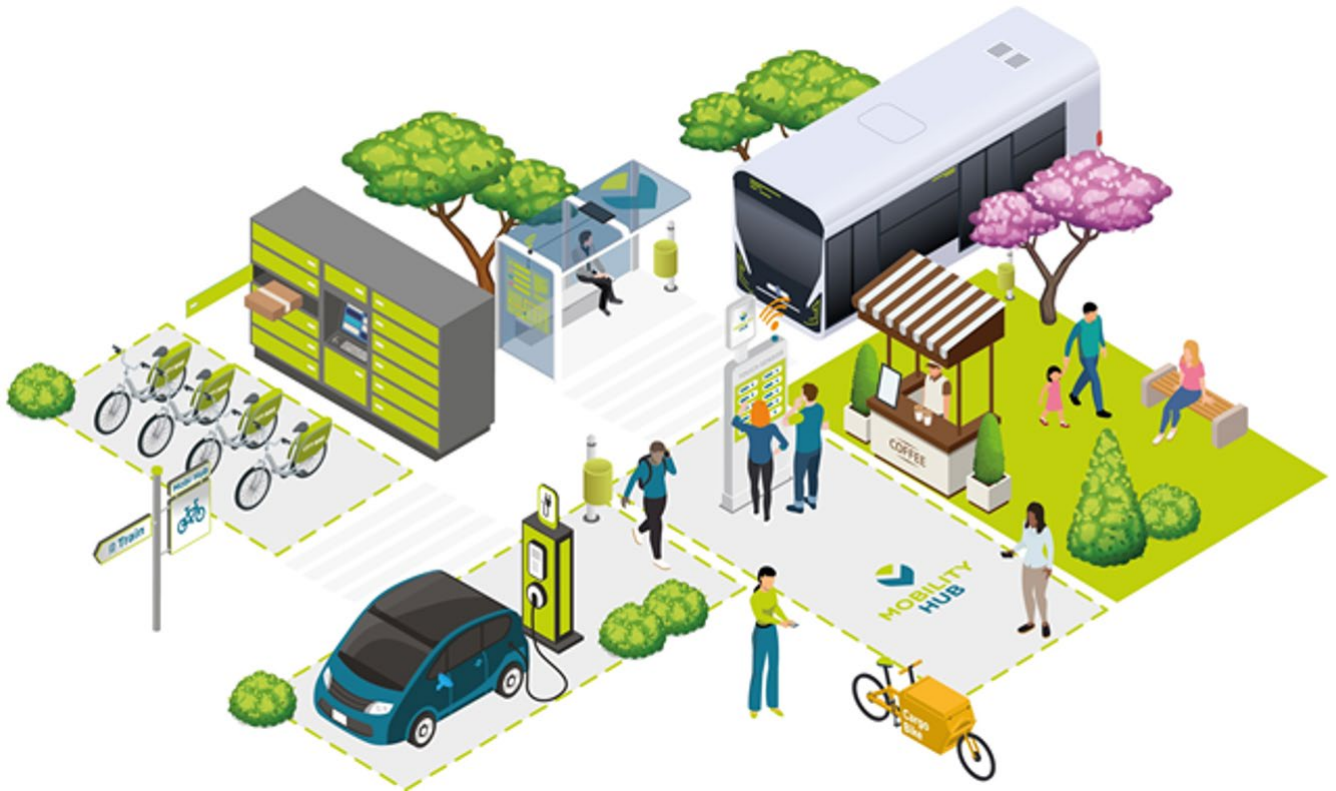
eHUBS schematic diagram^17^.

The eHUBS project, funded by Interreg North-West Europe in 2019, seeks to advance sustainable urban transport by promoting shared electric mobility (Fig. 1). E-mobility hubs (eHUBS) are designated on-street locations offering at least two shared electric modes, such as e-cars, e-bikes, e-cargo bikes, and e-scooters^17^. These hubs encourage residents to adopt shared electric transport as an alternative to traditional travel, fostering cleaner and more liveable cities. The project includes nine pilot cities across six countries where eHUBS were implemented and promoted: Amsterdam, Arnhem, and Nijmegen (Netherlands), Leuven (Belgium), Greater Manchester and Inverness (UK), Kempten (Germany), Dublin (Ireland), and Dreux (France)^17^.

As part of the eHUBS project, a comprehensive survey was conducted in Greater Manchester from 2021 to 2023, collecting a total of 1,220 travel records, of which 1,139 were valid. These data were used to assess the potential of eHUBS as an alternative to conventional travel modes and to evaluate the likelihood of eHUBS replacing conventional travel modes after implementation, with a focus on its impact on private car travel, public transport, and zero-carbon travel (i.e., walking and traditional cycling). For a broader overview of the eHUBS project and its methodological background, please refer to Bösehans et al. (2023b)^18^.

This study aims to quantify carbon emissions reduction through mode substitution enabled by eHUBS shared mobility services, based on survey results. The primary objectives are: (1) to analyse mode-switching behaviour among potential eHUBS users, (2) to establish a method for assessing the impact of e-mobility substitution on carbon emissions, (3) to quantify the carbon reduction benefits of shared e-mobility options, and (4) to offer recommendations on the sustainability of shared mobility as a viable alternative to current travel modes.

Despite growing interest in shared electric mobility, key research gaps persist. Existing studies largely overlook multi-modal eHUBS, lack empirical analysis of mode substitution based on observed behaviour, and insufficiently explore carbon reduction potential when integrated with public transport. This study addresses these gaps through a combined approach of behavioural analysis, substitution modelling, and carbon impact assessment.

To address these research gaps, this study investigates the substitution effects of eHUBS in Greater Manchester, assesses their integration with public transport, and quantifies the resulting carbon reduction. The proposed methodology is designed to be transferable to similar datasets and applicable in other urban contexts.

The structure of this paper is as follows: Sect. 2 reviews the literature on shared electric mobility and eHUBS related to mode substitution and carbon emissions reduction. Section 3 analyses the potential for eHUBS to replace conventional travel modes in Greater Manchester. Section 4 quantifies the impact of this substitution on carbon emissions reduction. Finally, Sect. 5 summarises the findings and offers recommendations.

## Literature review

This section systematically reviews the impacts of shared e-bikes, e-cargo bikes, e-scooters, and e-cars on replacing conventional travel modes and reducing carbon emissions, followed by an in-depth analysis of the combined effects of electric mobility hubs integrating these modes.

### Shared e-bike

Early studies on e-bike sharing largely emphasised its limited potential to replace conventional travel modes, often overlooking its role in reducing carbon emissions and focusing narrowly on specific locations. For instance, North America’s first e-bike sharing system was investigated at the University of Tennessee, Knoxville, finding that only 11% of e-bike trips replaced car journeys, with limited impact on regular bicycle trips, but 58% substituted pedestrian trips^19^. Conversely, a study conducted in Beijing found that e-bike sharing was preferred for substituting long-distance bus trips, with an average trip distance of 4.5 km, indicating its suitability for longer journeys compared to traditional bicycle sharing^20^. The study also identified several factors influencing the substitution of automobility with e-bike sharing, including user demographics, travel behaviour, system design, and environmental conditions^20^. A study in Tricity^8^ concluded that e-bike sharing does not significantly challenge car or bicycle use. More recent studies have expanded the focus to examine both the modal substitution and carbon reduction potential of shared e-bikes. In urban settings, e-bike sharing was found to largely replace car trips but had limited integration with public transit, with emissions of 19.47 g$\:{CO}_{2}$/km, including 6.91 g/km from electricity and 12.56 g/km from battery swapping and bike relocation^21^. In Chengdu, shared e-bikes were found to primarily reduce public transit and regular bicycle use rather than private car trips. Nevertheless, overall emissions decreased, with reductions from lowered transit use outweighing emissions from battery-related activities by 0.108–0.120 g/km^22^. By 2030, e-bike sharing in Changsha is projected to substitute 20% of car trips, 17% of taxi trips, 2.6% of bus trips, and 5.3% of metro trips, contributing to carbon reductions of 51.27%, 1.54%, 0.7%, and − 1.39%, respectively^23^. Despite occasional conflicting findings, numerous studies affirm the significant potential of shared e-bikes to reduce car dependency and lower carbon emissions, highlighting their viability as a sustainable urban transport solution.

### Shared e-cargo bike

Research on e-cargo bike sharing’s mode substitution and carbon reduction potential has gained attention in recent years. E-cargo bikes have been found to replace up to 10% of vans within a 2 km radius without reducing transport efficiency, cutting carbon emissions by as much as 73% for urban deliveries^24^. A study reported that 46% of respondents would switch to car use if e-cargo bike sharing were unavailable, underscoring the potential of such schemes to reduce car dependency and lower emissions by 166 g/km^13^. In London, e-cargo bikes were shown to be capable of replacing 10%–30% of trips, contributing to emission reductions and improved air quality^25^. Similarly, e-cargo bikes were found to be capable of substituting 68% of car-based and 55% of van-based deliveries, especially for short distances, significantly reducing emissions for small-scale services such as takeaways^26^. Studies have highlighted that e-cargo bike sharing differs functionally from e-bike sharing, as its capacity to carry goods and children results in more frequent use, especially among upper-middle-class men with family duties^27^. This functional differentiation reinforces the role of e-cargo bikes in substituting regular car trips for household and logistical purposes. Collectively, these studies highlight e-cargo bikes’ capacity to decrease car dependency and lower carbon emissions.

### Shared e-scooter

The discussion on mode substitution and carbon reduction related to e-scooter sharing has emerged relatively recently. In New Zealand, 57% of e-scooter trips were found to replace walking, biking, skateboarding, or e-biking, while 28% substituted motorised modes such as cars and taxis^28^. In Rosslyn, US, e-scooters were found to replace 39% of taxi trips, 33% of walks, 12% of bike rides, and 7% each of bus and car journeys^29^. However, neither study assessed the carbon emissions impact of these substitutions. Moreover, shared e-scooters have been argued to increase $\:{CO}_{2}$ emissions (approximately 51 g $\:{CO}_{2}$/km) relative to the modes they replace, particularly when substituting walking and public transport^30^. In Germany, over 60% of e-scooter trips were found to substitute walking and public transport, while only 11.5% replaced private bikes or cars. It was noted that current emissions outweigh reductions, although this trend could reverse in the future^31^. In Spain, shared e-scooters were found to substitute 56.5% of walking trips but only 2.1% of car trips^32^. Based on a comprehensive review of recent studies, it was concluded that e-scooters have limited potential to substitute public transport trips, primarily due to differing trip characteristics^33^. In Norway, e-scooters were found to frequently substitute for public transport when transit trips were long in duration, while also serving a complementary role in approximately 20% of multi-modal trips^34^. These findings highlight that the substitution effects of e-scooters on public transport vary significantly depending on urban context, infrastructure, and service integration. Therefore, the relationship between e-scooter use and public transport cannot be generalised in a singular way; instead, region-specific analysis is required to better understand their role in sustainable urban mobility.

### Shared e-car

Most studies on shared e-cars’ impact on conventional travel mode shifts approach this indirectly, examining current travel patterns, subjective intentions, and external conditions, likely due to early-stage technology and infrastructure limitations. The integration of e-car sharing within Berlin’s public transport system was first assessed through the Berlin elektroMobil project, which found improvements in user experience and encouraged mode shifts, although challenges remained regarding charging infrastructure and cost^15^. Acceptance of e-car sharing among rural residents in Berlin and Garmisch-Partenkirchen was found to be comparable to that of urban users^35^. In the Netherlands, e-car sharing was found to be primarily driven by social influence, with high satisfaction and trust boosting usage intentions, while private car ownership reduced them^36^. Although few studies have quantified shared e-cars’ potential for reducing transport emissions, evidence indicates their positive impact on sustainable transport, as users tend to reduce overall car use^37^. Despite the rising popularity of shared e-cars, research remains limited on how they influence travel mode shifts and carbon reduction, which is particularly relevant in Greater Manchester, where a shared e-car club has been established^38^.

### eHUBS and integrated systems

In recent years, research on e-mobility hubs (eHUBS) has developed, particularly focusing on user preferences and travel behaviours. A survey conducted across five European countries used multiple linear regression to identify factors influencing the adoption of eHUBS among both users and non-users^39^. The study found that a positive attitude towards shared mobility was the strongest predictor, but also noted that while eHUBS attract diverse user groups, their potential to replace private car use is limited. A detailed case study in Amsterdam employed Everett Rogers’ Diffusion of Innovations (DOI) theory^40^ using a representative population sample to identify potential users^18^. They found that individuals with higher education and in a no-car household, as well as families with multiple cars and children, are more likely to use eHUBS. A study explored the willingness to use eHUBS shared electric vehicles for both commuting and shopping purposes^41^. The study revealed that while half of the respondents were open to using shared electric vehicles, this willingness dropped significantly when combined with public transport^41^. Additionally, mode shifts were limited, with car drivers preferring shared electric cars and cyclists favouring e-bikes and e-cargo bikes^41^. To evaluate eHUBS network performance in Inverness, a novel method was applied. The results showed that ridership demand is significantly affected by population density and weather, implying that e-bikes are more appropriate for longer-distance travel than for complementing public transport^42^. Additionally, an investigation of eHUBS mode substitution in Amsterdam revealed that users prefer different shared modes depending on travel-related factors^43^. Specifically, public transport users are more likely to switch to eHUBS than car users, while cyclists and walkers, though initially hesitant, are more likely to use eHUBS for longer trips^43^. Despite these studies exploring user preferences and the impact of eHUBS on travel behaviours, there remains a lack of research on the consequential effects on the reduction of transport-related carbon emissions of promoting the use of eHUBS as an alternative to traditional travel modes. Therefore, this research gap highlights the necessity for further studies to comprehensively understand the broader environmental impacts of eHUBS which was the motivation for this paper.

### Research gaps

Table 1 summarises the literature referenced, categorising studies by their contributions to mode substitution and carbon emission reduction. Most research focuses on individual shared electric mobility modes and their role in mode substitution, often overlooking the potential of more sustainable, combined options encouraged in eHUBS cities. While some studies examine eHUBS, quantitative analysis on carbon reduction in mode substitution contexts remains limited, and the impact on local public transport use is largely unexplored. This paper addresses these gaps by investigating the potential of eHUBS to replace conventional travel modes and their broader implications for transport sustainability.

**Table 1:** Summary of previous related studies.

Reference number	e-mobility sharing	Case study location	Mode substitution	$\:{CO}_{2}$ reduction quantification
^8^		Tricity, Poland.	✓	×
^19^	*e-bike*	Knoxville, US	✓	×
^20^		Beijing, PRC	✓	×
^21^		Shanghai, PRC	✓	✓
^22^		Chengdu, PRC	✓	✓
^23^		Changsha, PRC	✓	✓
^13^	*e-cargo bike*	Germany	✓	✓
^24^		Porto, Portugal	✓	✓
^25^		London, UK	✓	✓
^26^		**-**	✓	✓
^27^		**-**	✓	×
^28^	*e-scooter*	New Zealand	✓	×
^29^		Rosslyn, US	✓	×
^30^		Zurich, Switzerland	✓	✓
^31^		Germany	✓	✓
^32^		Madrid, Spain	✓	×
^33^		**-**	✓	×
^34^		Norway	✓	×
^15^	*e-car*	Berlin, Germany	✓	×
^35^			✓	×
^36^		Netherlands, NL	✓	×
^37^		**-**	✓	✓
^18^	*eHUBS*	Amsterdam, NL	✓	×
^41^		7 EU cities	✓	×
^42^		Inverness, UK	×	×
^43^		Amsterdam, NL	✓	×
^44^		5 EU cities	✓	×

### Mode substitution

This section details data collection, preprocessing, and the analysis of eHUBS substitution potential for conventional modes. Electric mobility refers to shared systems unless specified.

### Survey implementation and data collection

This study investigates the impact of promoting the eHUBS model on travel mode shifts and carbon emission reductions in Greater Manchester. As part of the eHUBS project, survey data were collected between 2021 and 2023, yielding 1,139 valid responses from 1,220 participants. The survey (Appendix A) comprised four sections: (1) respondent demographics; (2) household vehicle characteristics (brand, model, fuel type); (3) daily trip information, including each travel segment and duration; and (4) the likelihood of adopting eHUBS modes (e-car, e-bike, e-cargo bike, e-scooter) as alternatives to conventional transport. Respondents evaluated two substitution scenarios, complete replacement and partial integration with public transport, using a five-point Likert scale (“I would not use it” to “I may use it for all trips”). These ordinal responses were initially mapped to probabilities of 0%, 25%, 50%, 75%, and 100%, following established practice in comparable studies (e.g., Zhou et al., 2022^21^. To assess the robustness of this mapping, Sect. 3.3 also briefly includes a sensitivity analysis employing a more conservative probability set (0%, 20%, 45%, 70%, 95%) to reflect a cautious interpretation of high-frequency adoption.

All procedures involving the design of data collection instruments were approved by Newcastle University’s SAGE Ethics Committee (Reference No. 18251/2019). The study was conducted in accordance with the Committee’s ethical standards, as well as the principles outlined in the 1964 Helsinki Declaration and its subsequent amendments or equivalent ethical guidelines. Informed consent for participation and, where applicable, for the publication of anonymised data and information in an online open-access format was obtained from all participants and/or their legal guardians.

This paper does not examine the impact of demographic factors on eHUBs substitution behaviour, as other eHUBs-related studies^18,41–44^ have already addressed this. Consequently, demographic data from the first part of the eHUBS survey was omitted here, with potential for future analysis. In the second survey section, 527 respondents reported the brand, model, and fuel type of their household vehicles. Notably, vehicle data from respondents who did not use a private car in their daily records were excluded, assuming these vehicles, although household-owned, were not primary for the respondents. The reported vehicle information was then categorised based on the European Commission’s 1999 car market segments^45^ (see Table 2), which classifies passenger cars by engine size or length. Table 2 reveals that most surveyed vehicles fall into segments A, B, and C, comprising 74.00% of the sample, suggesting that Greater Manchester residents largely own small to medium-sized, fuel-efficient cars.

**Table 2:** Vehicle brand, model, and fuel type of respondents with private car usage records in greater Manchester. (Note: PHEV stands for plug-in hybrid electric vehicle.)

Segment	No.	Fuel type	No.	$\:\%$	Segment	No.	Fuel type	No.	$\:\%$
*A-Mini*: 12.33%	65	Petrol	63	96.92	*E-Executive*: 1.33%	7	Petrol	1	14.30
		Diesel	2	3.08			Diesel	4	57.10
		PHEV	0	0.00			PHEV	0	0.00
		Electric	0	0.00			Electric	2	28.60
*B-Small*: 33.21%	175	Petrol	154	88.00	*J-Sport utility* 7.78%	41	Petrol	17	41.46
		Diesel	17	9.71			Diesel	17	41.46
		PHEV	1	0.57			PHEV	7	17.07
		Electric	3	1.71			Electric	0	0.00
*C-Medium*: 28.46%	150	Petrol	90	60.00	*M-multi-purpose*: 7.02%	37	Petrol	20	54.05
		Diesel	54	36.00			Diesel	15	40.54
		PHEV	4	2.67			PHEV	2	5.41
		Electric	2	1.33			Electric	0	0.00
*D-Large*: 7.21%	38	Petrol	16	42.11	*S-Sports*: 2.66%	14	Petrol	7	50.00
		Diesel	18	47.37			Diesel	7	50.00
		PHEV	2	5.26			PHEV	0	0.00
		Electric	2	5.26			Electric	0	0.00

In the third section, based on valid travel records, seven conventional travel modes are summarised: private cars, taxis, motorbikes, buses, light rail transit (LRT), private bicycles, and walking. These modes are grouped into three categories based on carbon emissions: motorised travel (CTM), including private cars, taxis, and motorbikes; public transport (PT), covering buses and LRT; and zero-carbon travel (WB), including walking and cycling. Typically, motorised travel has the highest carbon emissions, public transport is moderate, and walking and cycling produce no emissions (excluding minimal dietary carbon intake for energy). It is important to note that this study does not include lifecycle emissions in the carbon calculations. Survey records also reveal frequent combinations of CTM and PT with WB, such as “private car + walking” or “LRT + cycling.” Thus, conventional travel modes in this study are defined as: (1) CTM + WB, (2) PT + WB, and (3) pure WB travel. These combinations serve as a foundation for analysing eHUBS substitution potential and carbon reduction impact. This study specifically evaluates the potential of eHUBS to replace CTM + WB combinations, explores integration with PT, and assesses the impact on existing WB travel.

### Mode substitution of eHUBS

For clarity and ease of reference, all notations used throughout this paper are summarised in Table 3. Note that $\:CO_{2}e$ is used throughout this paper to express greenhouse gas emissions. It refers to a standardised metric that converts the impact of various greenhouse gases into an equivalent amount of carbon dioxide based on their global warming potential (GWP).

**Table 3:** Notations.

Indexes	
$\:i$	Conventional travel modes combinations, 1:“CTM + WB”, 2:“PT + WB”, 3: “WB”
$\:j$	Distance interval, 1: “[0 km,5 km)”, 2: “[5 km,10 km)”, 3: “[10 km, 20 km)”, 4: “[20 km,$\:+\infty\:)$
$\:k$	eHUBS travel modes, 1: “e-car”, 2: “e-bike”, 3: “e-cargo bike”, 4: “e-scooter”
$\:s$	Conventional travel modes, 1: “private cars”, 2: “taxis”, 3: “motorbikes”, 4: “buses”, 5: “LRT”, 6: “cycling”, and 7: “walking”
Events	
$\:{E}_{k}$	The event where the user substitutes $\:i$ with $\:k$ in Greater Manchester.
$\:{F}_{k}$	The event where mode $\:k$ is chosen with public transport to partially substitute conventional travel mode $\:i$ in Greater Manchester.
$\:{G}_{i}$	The event where the user selects $\:i$ for a specific trip in Greater Manchester.
$\:{H}_{j}$	The event where the user’s travel distance falls within distance interval $\:j$ in Greater Manchester.
$\:P\left(\right)$	Probability of an event
Other parameters	
$\:{C}_{s}$	Carbon emission factor of conventional travel mode $\:s$.
$\:{C}_{k}$	Carbon emission factor of eHUBS travel mode $\:k$.
$\:{d}_{ij}$	The distance per trip within distance interval $\:j$ for mode combination $\:i$.
$\:{D}_{ij}$	The total travel distance within distance interval $\:j$ for mode combination $\:i$.
$\:{l}_{kij}$	The distance conversion factor within $\:j$, adjusting the travel distance from $\:i$ to $\:k$ when substituting for the same journey with identical origin and destination.
$\:N$	Trip number, where $\:{N}_{i}$ is the number of trips using mode combination $\:i$, while $\:{N}_{ij}$ is the number of trips using $\:i$ within travel interval $\:j$.
$\:{r}_{j}$	Within interval $\:j$, the average proportion of WB distance relative to the total travel distance $\:D$ in the PT + WB travel mode combination.
$\:{t}_{s}$	The time (h) spent by a traveller using travel mode $\:s$ during a single trip.
$\:{T}_{kij}$	The number of trips substituting $\:i$ with $\:k$ within $\:j$.
$\:\overline{{v}_{s}}$	The average speed of conventional travel mode $\:s$ in the Greater Manchester.
Results	
$\:{M}_{kij}$	The carbon emission generated by mode $\:k$ under the eHUBS substitution effect.
$\:{\varDelta\:M}_{kj}$	The contribution of the four eHUBS travel mode $\:k$ to $\:CO_{2}e$ reduction in travel interval$\:\:j$ under both whole-trip and partial-trip substitution scenarios
$\:{q}_{ij}$	The average $\:CO_{2}e$ produced by the trips in mode combination $\:i$ within interval $\:j$ prior to the introduction of eHUBS.
$\:{Q}_{ij}$	The aggregate carbon emissions produced by the trips in mode combination $\:i$ within interval $\:j$ prior to the introduction of eHUBS.
$\:\varDelta\:{Q}_{ij}$	Changes in $\:CO_{2}e$ for the total trips within mode combination $\:i$ and interval $\:j$, comparing scenarios with and without the adoption of eHUBS.

To evaluate the effectiveness of eHUBS in substituting conventional travel modes, this study adopts a structured three-step methodology grounded in Bayes’ Theorem. Each analytical step is visualised through a tabular workflow (see Table 4), which systematically illustrates the introduction and interaction of key probabilistic parameters.

**Table 4:** Structured tabular workflow of the three-step methodology for estimating mode substitution effects of eHUBS, based on bayes’ Theorem.

Step	Step purpose	Key formula
Step I. Baseline joint probability without eHUBS	Determine the joint probability of a random trip being of travel interval $\:j$ and taken by a conventional travel mode combination $\:i$ when eHUBS mode $\:k$ are unavailable.	$\:P\left({G}_{i}{H}_{j}\right) = P\left({G}_{i}|{H}_{j}\right)P\left({H}_{j}\right)$
Step II. Joint probability of substitution with eHUBS	Determine the joint probability of a random trip in distance interval $\:j$, originally using mode combination $\:i$, being substituted by eHUBS mode $\:k$ when available.	$\:P\left({E}_{k}{G}_{i}{H}_{j}\right) = \tilde{P}\left({E}_{k}\right|{G}_{i}{H}_{j})P\left({G}_{i}{H}_{j}\right)$
Step III. Conditional attribution of original mode	Determine the conditional probability that, within distance interval $\:j$, a journey substituted by eHUBS mode $\:k$ (both partially or fully) would have originally been made using mode combination $\:i$ if eHUBS mode $\:k$ were unavailable.	$\:P\left({E}_{k}{G}_{i}|{H}_{j}\right) = \frac{P\left({E}_{k}{G}_{i}{H}_{j}\right)}{P\left({H}_{j}\right)}$ $\:P\left({F}_{k}{E}_{k}{G}_{i}|{H}_{j}\right) = \frac{P\left({F}_{k}{E}_{k}{G}_{i}{H}_{j}\right)}{P\left({H}_{j}\right)}$ $\:P\left[(1-{F}_{k}){E}_{k}{G}_{i}|{H}_{j}\right] = P\left({E}_{k}{G}_{i}|{H}_{j}\right)-P\left({F}_{k}{E}_{k}{G}_{i}|{H}_{j}\right)$

*Step I*: Based on the 1,139 reported trips, the probability $\:P\left({H}_{j}\right)$ is determined (see Table 5), representing the likelihood of selecting any conventional travel mode combination $\:i\:$within four different travel distance intervals in the absence of eHUBS. These probabilities are denoted as $\:P\left({H}_{1}\right)$, $\:P\left({H}_{2}\right)$, $\:P\left({H}_{3}\right)$, and $\:P\left({H}_{4}\right)$, corresponding to short-distance, medium-short-distance, medium-long-distance, and long-distance trips, respectively. Next, $\:P\left({G}_{i}\right|{H}_{j})$ ($\:i$ = 1,2,3) is decided, representing the conditional probability of selecting mode combination $\:i$ given travel distance $\:j$ in the absence of eHUBS. Please note that within the PT + WB travel mode combination, 22 records involved the CTM + PT + WB combination. These records typically indicate that individuals first travel by car to a public transport hub, then use public transport to reach a nearby destination, and finally walk to their destination. However, due to the small sample size of this combination and the short car travel times (usually within 5 min), it is primarily considered as PT + WB, with public transport being the main mode. Then, based on Bayes’ Theorem (Eq. 1), $\:P\left({G}_{i}{H}_{j}\right)$ is derived (see Table 5), representing the joint probability of a randomly selected trip covering distance interval $\:j$ and utilising conventional travel mode combination $\:i$ when eHUBS is unavailable. For example, $\:P\left({G}_{1}{H}_{1}\right)$ = 13.7% indicates that in Greater Manchester, the joint probability of randomly selecting the CTM+WB combination for trips within the [0, 5 km) range is 13.7%.

$$\:P\left({G}_{i}{H}_{j}\right) = P\left({G}_{i}|{H}_{j}\right)P\left({H}_{j}\right)$$1.

**Table 5:** Probabilities of distance interval $\:P\left({H}_{j}\right)$, conditional mode choice $\:P\left({G}_{i}\right|{H}_{j})$, and joint probability $\:P\left({G}_{i}{H}_{j}\right)$ in the absence of eHUBS.

$\:{H}_{j}$	$\:{N}_{j}$	$\:P\left({H}_{j}\right)\:$(%)	$\:{G}_{i}$	$\:{N}_{ij}$	$\:P\left({G}_{i}\right|{H}_{j})$ (%)	$\:P\left({G}_{i}{H}_{j}\right)$ (%)
$\:{H}_{1}$	441	38.72%	$\:{G}_{1}$	156	35.37%	13.70%
			$\:{G}_{2}$	34	7.71%	2.98%
			$\:{G}_{3}$	251	56.92%	22.04%
$\:{H}_{2}$	296	25.99%	$\:{G}_{1}$	235	79.39%	20.63%
			$\:{G}_{2}$	53	17.91%	4.66%
			$\:{G}_{3}$	8	2.70%	0.70%
$\:{H}_{3}$	226	19.84%	$\:{G}_{1}$	168	74.34%	14.75%
			$\:{G}_{2}$	53	23.45%	4.65%
			$\:{G}_{3}$	5	2.21%	0.44%
$\:{H}_{4}$	176	15.45%	$\:{G}_{1}$	147	83.52%	12.90%
			$\:{G}_{2}$	28	15.91%	2.46%
			$\:{G}_{3}$	1	0.57%	0.09%

*Step II* To determine the joint probability $\:P({E}_{k}{G}_{i}{H}_{j}$) $\:(k = \text{1,2},\text{3,4})$ of a randomly selected trip transitioning from a conventional travel mode combination$\:\:i$ to an eHUBS mode $\:k$ within a given travel distance interval $\:j$ when eHUBS are available, $\:P\left({E}_{k}\right|{G}_{i}{H}_{j})$ is obtained, which represents the conditional probability of transitioning to eHUBS mode $\:k$ given that the trip is originally undertaken using conventional mode combination $\:i$ and covered a distance of $\:j$. Specifically, $\:P\left({E}_{k}|{G}_{i}{H}_{j}\right),\:(k = \text{1,2},\text{3,4})$ represent the conditional probabilities of respondents being willing to use an e-car, e-bike, e-cargo bike, and e-scooter, respectively, to substitute their original travel mode combination $\:i$. According to the final section of the survey and by calculating the average value of $\:P\left({E}_{k}\right|{G}_{i}{H}_{j})$ across each distance interval for different mode combination $\:i$, $\:\overline{P}\left({E}_{k}\right|{G}_{i}{H}_{j})$ is obtained. Moreover, to preserve the mutual exclusivity between transitioning from conventional mode combination $\:i$ to the eHUBS mode $\:k$ and continuing with combination $\:i$, $\:\overline{P}\left({E}_{k}\right|{G}_{i}{H}_{j})$ is normalised by setting $\:{\sum\:}_{k = 1}^{4}\tilde{P}\left({E}_{k}|{G}_{i}{H}_{j}\right)+{\sum\:}_{k = 1}^{4}\tilde{P}\left[\left(1-{E}_{k}\right)|{G}_{i}{H}_{j}\right]$ = 1, where $\:\tilde{P}\left({E}_{k}|{G}_{i}{H}_{j}\right)$ is denoted as the normalised probability. The results for $\:\overline{P}\left({E}_{k}\right|{G}_{i}{H}_{j})$ and $\:\tilde{P}\left({E}_{k}\right|{G}_{i}{H}_{j})$ are concluded in Appendix B.

Next, $\:P\left({E}_{k}{G}_{i}{H}_{j}\right)$ is calculated using Eq. 2 (Please see details from Table I to Table IV in Appendix B), representing the joint probability of a randomly selected trip with distance interval $\:j$ in Greater Manchester, originally undertaken by conventional travel mode combination $\:i$, transitioning to eHUBS mode $\:k$. The results of $\:P\left({E}_{k}{G}_{i}{H}_{j}\right)$ are summarised in Appendix B. For example, according to Table I in Appendix B, $\:P\left({E}_{1}{G}_{1}{H}_{1}\right)$ = 1.20% indicates that in Greater Manchester, the joint probability of randomly selecting an individual whose trip distance falls within the [0, 5 km) interval, who originally used the CTM+WB travel mode combination but wants to switch to using an e-car, is 1.20%.

$$\:P\left({E}_{k}{G}_{i}{H}_{j}\right) = \tilde{P}\left({E}_{k}\right|{G}_{i}{H}_{j})P\left({G}_{i}{H}_{j}\right)\:$$2.

*Step III* Equation 3 is employed to calculate the conditional probability $\:P\left({E}_{k}{G}_{i}|{H}_{j}\right)$ that a traveller, originally using mode combination $\:i$ within the distance interval $\:j$, is inclined to switch to eHUBS mode $\:k$ (see Table 6). For example, $\:P\left({E}_{1}{G}_{1}\right|{H}_{1})$ = 3.1% indicates that in Greater Manchester, within the travel distance range of [0, 5 km), the conditional probability that a traveller originally using the CTM+WB would be willing to switch to using e-car is 3.1%.

$$\:P\left({E}_{k}{G}_{i}|{H}_{j}\right) = \frac{P\left({E}_{k}{G}_{i}{H}_{j}\right)}{P\left({H}_{j}\right)}$$3.

**Table 6:** Conditional probabilities $\:P\left({E}_{k}{G}_{i}\right|{H}_{j}$): the likelihood of switching from mode combination $\:i$ to eHUBS mode $\:k$ within distance interval $\:j$.

$\:{H}_{j}$	$\:{G}_{i}$	$\:P(.)$ (%)	$\:k$ = 1	$\:k$ = 2	$\:k$ = 3	$\:k$ = 4	$\:{\sum\:}_{k = 1}^{4}P\left({E}_{k}{G}_{i}{H}_{j}\right)$	$\:{\sum\:}_{k = 1}^{4}P\left[\left(1-{E}_{k}\right){G}_{i}{H}_{j}\right]$
$\:{H}_{1}$	$\:{G}_{1}$	$\:{E}_{k}{G}_{1}|{H}_{1}$	3.10	3.69	2.17	2.74	11.70	23.66
	$\:{G}_{2}$	$\:{E}_{k}{G}_{2}|{H}_{1}$	0.21	1.03	0.80	0.65	2.69	5.04
	$\:{G}_{3}$	$\:{E}_{k}{G}_{3}|{H}_{1}$	1.60	6.35	4.47	4.36	16.78	40.13
$\:{H}_{2}$	$\:{G}_{1}$	$\:{E}_{k}{G}_{1}|{H}_{2}$	7.58	6.47	4.74	5.62	24.41	54.99
	$\:{G}_{2}$	$\:{E}_{k}{G}_{2}|{H}_{2}$	0.62	1.50	2.08	1.35	5.55	12.34
	$\:{G}_{3}$	$\:{E}_{k}{G}_{3}|{H}_{2}$	0.04	0.27	0.16	0.16	0.63	2.08
$\:{H}_{3}$	$\:{G}_{1}$	$\:{E}_{k}{G}_{1}|{H}_{3}$	7.36	5.70	6.90	6.25	26.21	48.14
	$\:{G}_{2}$	$\:{E}_{k}{G}_{2}|{H}_{3}$	1.76	1.66	2.57	1.97	7.96	15.47
	$\:{G}_{3}$	$\:{E}_{k}{G}_{3}|{H}_{3}$	0.10	0.15	0.10	0.05	0.40	1.81
$\:{H}_{4}$	$\:{G}_{1}$	$\:{E}_{k}{G}_{1}|{H}_{4}$	8.54	3.11	7.57	5.76	24.98	58.55
	$\:{G}_{2}$	$\:{E}_{k}{G}_{2}|{H}_{4}$	1.49	1.36	1.75	2.00	6.60	9.30
	$\:{G}_{3}$	$\:{E}_{k}{G}_{3}|{H}_{4}$	0.00	0.00	0.00	0.00	0.00	0.56

To determine the conditional probability that eHUBS mode $\:k$ either wholly or partially substitutes a trip originally undertaken by conventional travel mode combination $\:i$ across the range of distance intervals $\:j$, two scenarios are defined: “whole-trip substitution” and “partial-trip substitution.” The first scenario indicates that eHUBS mode $\:k$ entirely replaces the conventional travel mode combination $\:i$. In contrast, in the second scenario, eHUBS mode $\:k$ replaces a portion of the journey, specifically the segment from the point of origin to the public transport connection, while the main part of the trip is completed via public transport.

Then, Eq. 4 is used to calculate the joint probability $\:P\left({F}_{k}{E}_{k}{G}_{i}{H}_{j}\right)$, representing the likelihood that eHUBS mode $\:k$ partially substitutes the conventional travel mode combination $\:i\:$within a specific distance interval $\:j$. In this context, $\:{F}_{k}$ denotes the event where users opt to partially use eHUBS mode $\:k$ while completing the remaining trip via public transport. The average conditional probability $\:\overline{P}\left({F}_{k}|{E}_{k}{G}_{i}{H}_{j}\right)$ is derived from the final section of the survey and reflects respondents’ willingness, within a given distance interval $\:j$, to integrate eHUBS with public transport as a substitute for their original travel mode combination $\:i$ in Greater Manchester. The results for $\:\overline{P}\left({F}_{k}|{E}_{k}{G}_{i}{H}_{j}\right)$ and $\:P\left({F}_{k}{E}_{k}{G}_{i}{H}_{j}\right)$ are detailed in Appendix B.

$$\:P\left({F}_{k}{E}_{k}{G}_{i}{H}_{j}\right) = \overline{P}\left({F}_{k}|{E}_{k}{G}_{i}{H}_{j}\right)P\left({E}_{k}{G}_{i}{H}_{j}\right)$$4.

Finally, using Eq. 5, $\:P\left({F}_{k}{E}_{k}{G}_{i}|{H}_{j}\right)$ is calculated, representing the conditional probability that a traveller, originally using conventional travel mode combination $\:i$ within distance interval $\:j$, is inclined to switch to eHUBS mode $\:k$ partially. All results for $\:P\left({F}_{k}{E}_{k}{G}_{i}|{H}_{j}\right)$ are summarised in Table 7. For example, $\:P\left({F}_{1}{E}_{1}{G}_{1}|{H}_{1}\right)$ = 1.2% indicates that, in Greater Manchester, the conditional probability of a traveller, within a travel distance of 5 km, originally using the CTM+WB, opting to switch to a combination of e-car and public transport is 1.2%. Moreover, using Eq. 6, the conditional probability of wholly replacing the original travel mode combination with an eHUBS mode is calculated.

$$\:P\left({F}_{k}{E}_{k}{G}_{i}|{H}_{j}\right) = \frac{P\left({F}_{k}{E}_{k}{G}_{i}{H}_{j}\right)}{P\left({H}_{j}\right)}$$5.

$$\:P\left[(1-{F}_{k}){E}_{k}{G}_{i}|{H}_{j}\right] = P\left({E}_{k}{G}_{i}|{H}_{j}\right)-P\left({F}_{k}{E}_{k}{G}_{i}|{H}_{j}\right)$$6.

**Table 7:** Conditional probabilities $\:P\left({F}_{k}{E}_{k}{G}_{i}|{H}_{j}\right)$: likelihood of partially substituting combination $\:i$ with eHUBS mode $\:k$ in combination with public transport, within interval $\:j$.

$\:{H}_{j}$	$\:{G}_{i}$	$\:P\left(.\right)$ %	$\:k = 1$	$\:k = 2$	$\:k = 3$	$\:k = 4$	$\:{\sum\:}_{k = 1}^{4}P\left[(1-{F}_{k}){E}_{k}{G}_{i}|{H}_{j}\right]$
$\:{H}_{1}$	$\:{G}_{1}$	$\:{F}_{k}{E}_{k}{G}_{1}|{H}_{1}$	1.18%	2.44%	0.72%	1.89%	6.23%
	$\:{G}_{2}$	$\:{F}_{k}{E}_{k}{G}_{2}|{H}_{1}$	0.07%	0.68%	0.53%	0.46%	1.74%
	$\:{G}_{3}$	$\:{F}_{k}{E}_{k}{G}_{3}|{H}_{1}$	0.21%	3.59%	1.77%	2.73%	8.30%
$\:{H}_{2}$	$\:{G}_{1}$	$\:{F}_{k}{E}_{k}{G}_{1}|{H}_{2}$	2.26%	4.07%	2.19%	3.41%	11.93%
	$\:{G}_{2}$	$\:{F}_{k}{E}_{k}{G}_{2}|{H}_{2}$	0.07%	0.96%	1.44%	0.86%	3.33%
	$\:{G}_{3}$	$\:{F}_{k}{E}_{k}{G}_{3}|{H}_{2}$	0.00%	0.14%	0.05%	0.03%	0.22%
$\:{H}_{3}$	$\:{G}_{1}$	$\:{F}_{k}{E}_{k}{G}_{1}|{H}_{3}$	1.69%	3.48%	3.82%	4.40%	13.39%
	$\:{G}_{2}$	$\:{F}_{k}{E}_{k}{G}_{2}|{H}_{3}$	0.37%	1.22%	1.81%	1.21%	4.61%
	$\:{G}_{3}$	$\:{F}_{k}{E}_{k}{G}_{3}|{H}_{3}$	0.01%	0.07%	0.02%	0.04%	0.14%
$\:{H}_{4}$	$\:{G}_{1}$	$\:{F}_{k}{E}_{k}{G}_{1}|{H}_{4}$	1.26%	2.09%	5.24%	3.97%	12.56%
	$\:{G}_{2}$	$\:{F}_{k}{E}_{k}{G}_{2}|{H}_{4}$	0.54%	1.00%	1.37%	1.18%	4.09%
	$\:{G}_{3}$	$\:{F}_{k}{E}_{k}{G}_{3}|{H}_{4}$	0.00%	0.00%	0.00%	0.00%	0.00%

### Analysis of mode substitution

This section presents Sankey diagrams (Figs. 2, 3, 4, 5, 6, 7, 8 and 9) illustrating eHUBS substitution probabilities across four distance intervals and analyses their substitution effect and integration with public transport.

#### eHUBS substitution effect

**Fig. 2:**
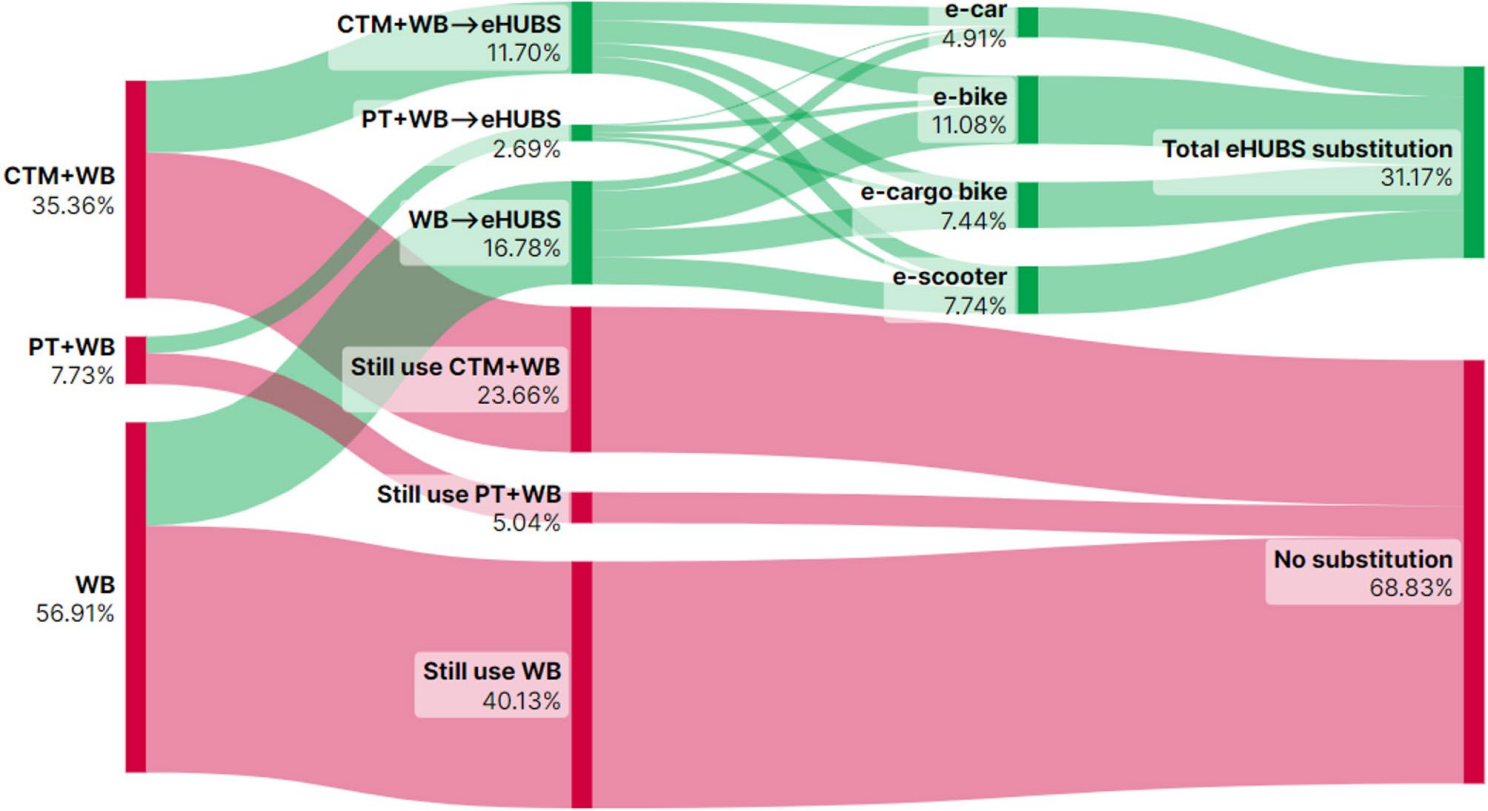
eHUBS substitution probability $\:P\left({E}_{k}{G}_{i}|{H}_{1}\right)$ within [0, 5 km). Generated using SankeyArt v1.4 (2025), https://www.sankeyart.com.

**Fig. 3:**
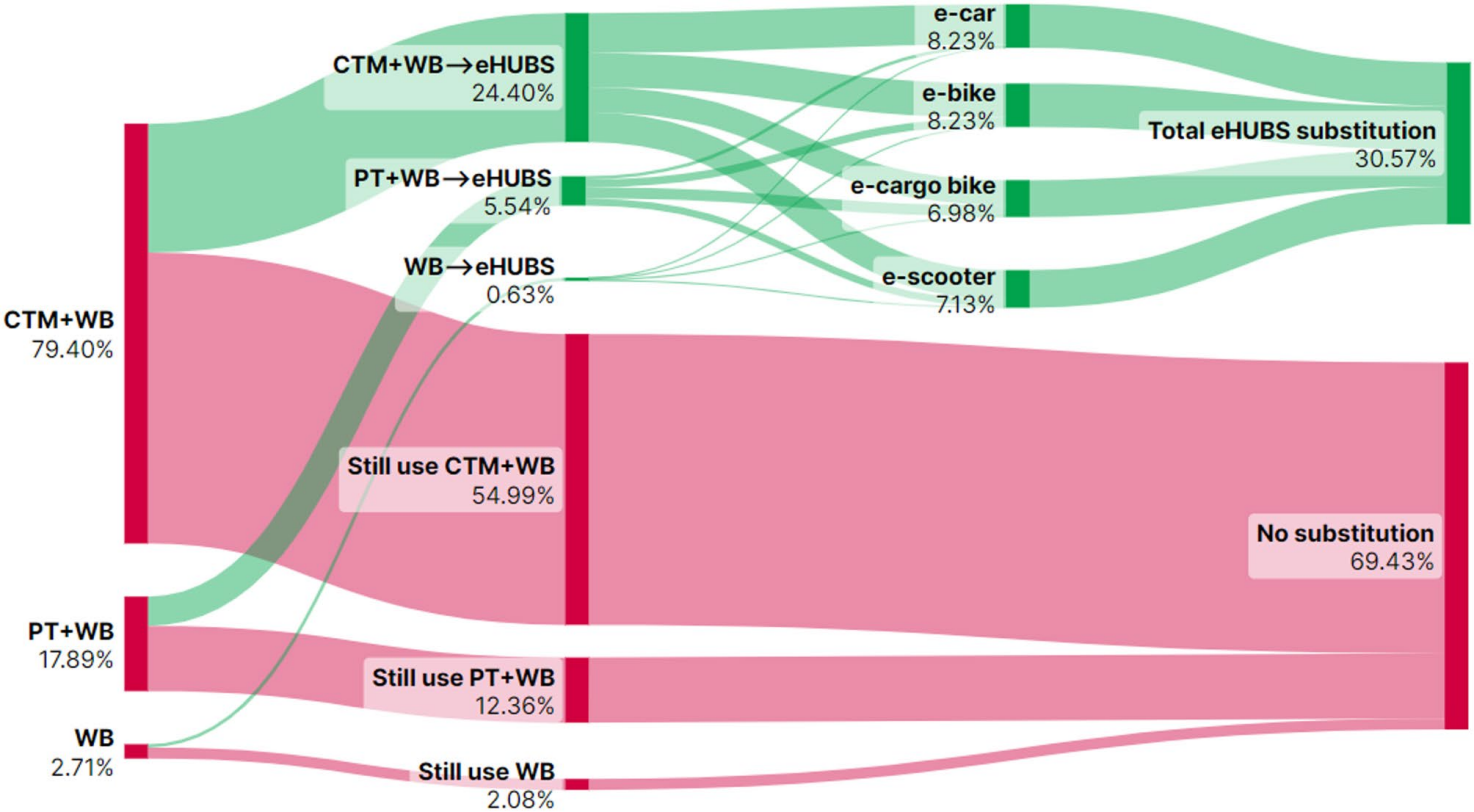
eHUBS substitution probability $\:P\left({E}_{k}{G}_{i}|{H}_{2}\right)$ within [5, 10 km). Generated using SankeyArt v1.4 (2025), https://www.sankeyart.com.

**Fig. 4:**
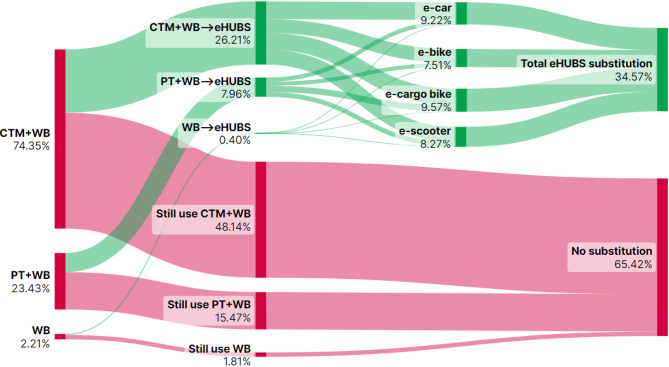
eHUBS substitution probability $\:P\left({E}_{k}{G}_{i}|{H}_{3}\right)$ within [10, 20 km). Generated using SankeyArt v1.4 (2025), https://www.sankeyart.com.

**Fig. 5:**
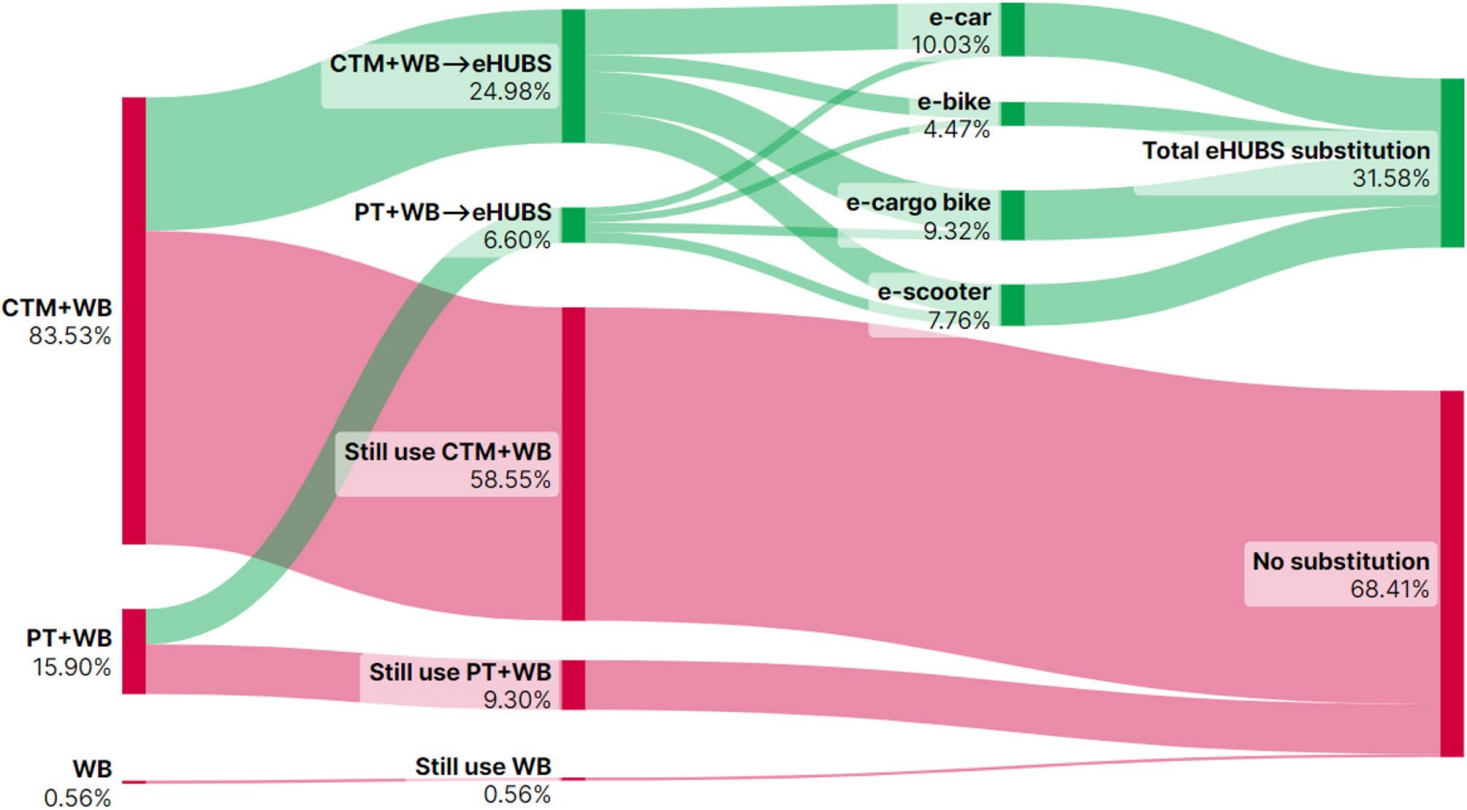
eHUBS substitution probability $\:P\left({E}_{k}{G}_{i}|{H}_{4}\right)\:$within [20 km, $\:+\infty\:$). Generated using SankeyArt v1.4 (2025), https://www.sankeyart.com.

The substitution effect of eHUBS varies across four travel distance intervals, with an overall substitution rate consistently between 30% and 35%. For short trips [0 km, 5 km), the substitution rate reaches 31.17% (Fig. 2), primarily replacing zero-carbon travel modes (walking and cycling), with shared e-bikes showing a particularly high rate of 11.08%. In the medium-short distance range [5 km, 10 km), the rate slightly decreases to 30.57% (Fig. 3), focusing more on replacing motorised travel combinations (CTM + WB), accounting for 24.40% of total substitutions. For medium-long distances [10 km, 20 km), the substitution rate increases to 34.57% (Fig. 4), with significant replacements of CTM + WB combinations, especially through the growing use of e-cargo bikes and e-cars. For long distances (above 20 km), the overall substitution rate is 31.58% (Fig. 5), with e-cars and e-cargo bikes showing substitution rates of 10.03% and 9.32%, respectively, reflecting their suitability for longer journeys. Overall, as travel distance increases, the eHUBS substitution effect shifts from short-distance replacement of walking and cycling to longer-distance substitution of motorised travel, demonstrating a preference for e-cars and e-cargo bikes.

A comparative analysis of the eHUBS substitution effect with other studies reveals several findings. For trips under 5 km, shared e-bikes in Greater Manchester replace CTM + WB and WB modes with a substitution rate over 11% (Fig. 2), accounting for one-third of overall eHUBS substitution in that range. This contrasts with Bielinski et al. (2021)^8^, whose Tricity study found that shared e-bikes had limited impact on replacing car or traditional bicycle use. However, our study similarly observes that as travel distance increases, the substitution effect of e-bikes on motorised, walking, and cycling modes declines, which aligns with the patterns identified in Bielinski et al. (2021)^8^. For trips over 10 km, e-cargo bikes show a substitution rate close to 10% (Figs. 4 and 5), partially supporting the previous findings^25^, while for trips below 10 km, the substitution rate of e-cargo bikes is lower, differing from their conclusions. Additionally, the substitution rate of e-scooters in this study remains consistently lower than those reported by the early studies^28,29^, respectively. These differences may arise from the following factors: (1) this study focuses on multiple shared electric modes within eHUBS, whereas most existing research examines single modes; and (2) this study provides a more granular analysis by considering substitution rates across various distance intervals.

#### eHUBS integration with public transport

**Fig. 6:**
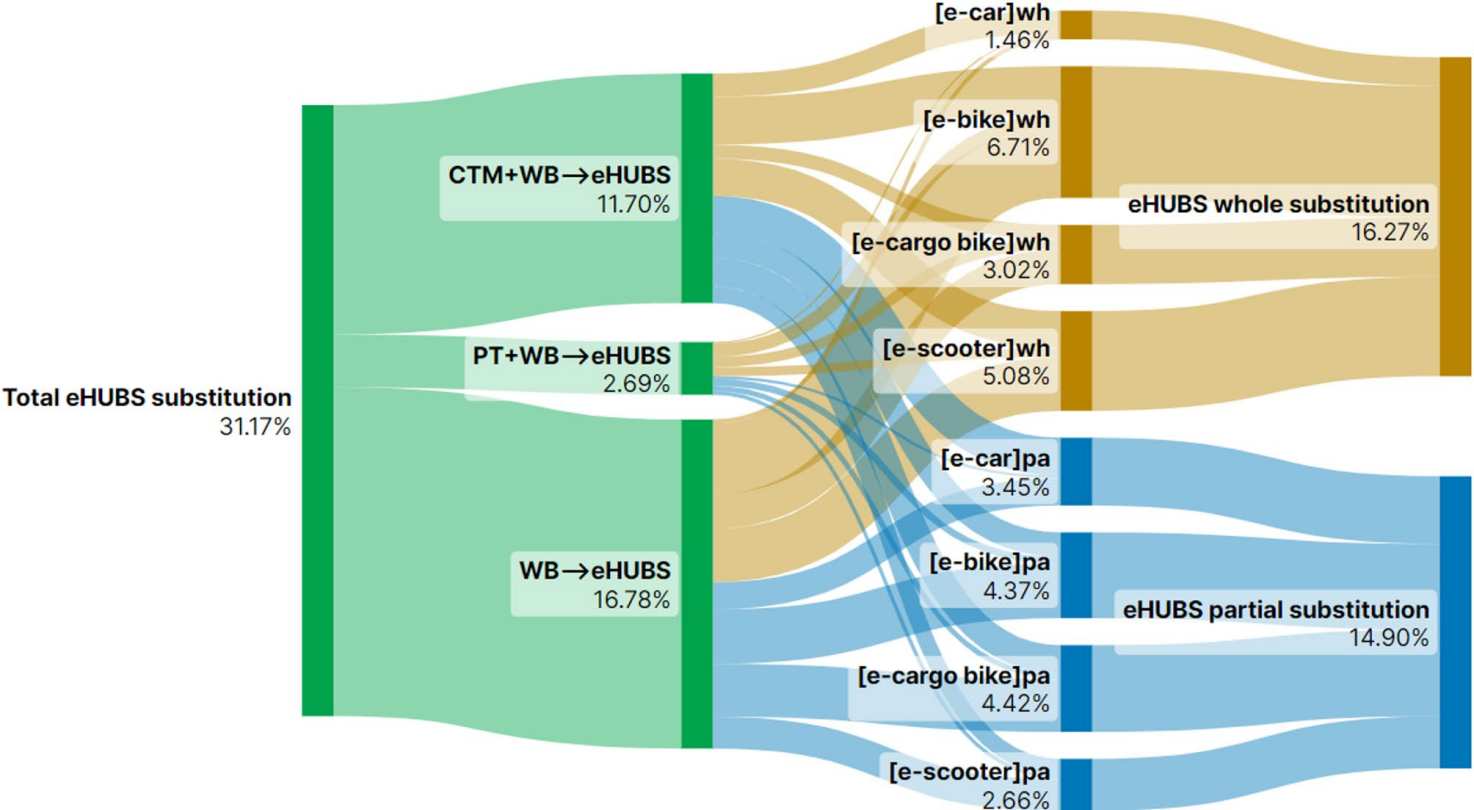
Substitution probabilities within [0, 5 km): whole vs. partial trips. Generated using SankeyArt v1.4 (2025), https://www.sankeyart.com.

**Fig. 7:**
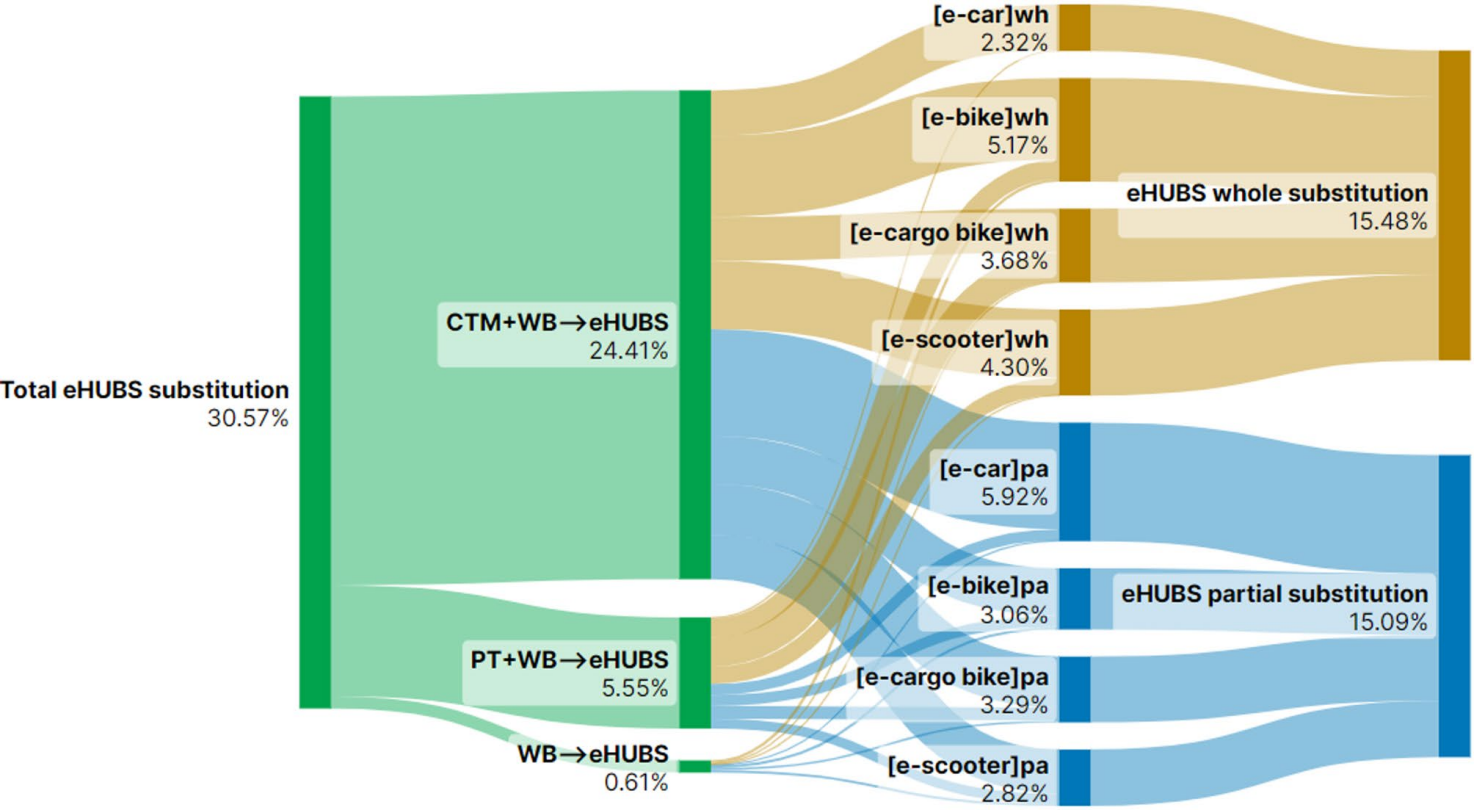
Substitution probabilities within [5, 10 km): whole vs. partial trips. Generated using SankeyArt v1.4 (2025), https://www.sankeyart.com.

Integration of eHUBS with public transport is evident across all travel distance intervals, with variations in extent by distance. In the shortest distance interval [0–5 km), 14.90% of users opt for partial-trip substitution by combining eHUBS with public transport (Fig. 6). Together with the 5.04% of users continuing to use the PT + WB combination (Fig. 2), this more than doubles the original proportion of users relying solely on public transport (7.73%, Fig. 2). This suggests that eHUBS, particularly via e-bikes (4.37%) and e-cargo bikes (4.42%), can effectively increase public transport use for short trips, though it is important not to overcomplicate these shorter journeys through excessive mode integration. In the medium-short range [5–10 km), eHUBS and public transport combinations increase slightly to 15.09% (Fig. 7). Including the 12.36% of users who continue with the PT + WB combination (Fig. 3), the overall proportion of public transport users rises by 9.56% from the original level (17.89%, Fig. 3). In this interval, e-cars (5.92%, Fig. 7) are increasingly preferred in conjunction with public transport, indicating a growing trend towards using eHUBS for moderately longer trips.

**Fig. 8:**
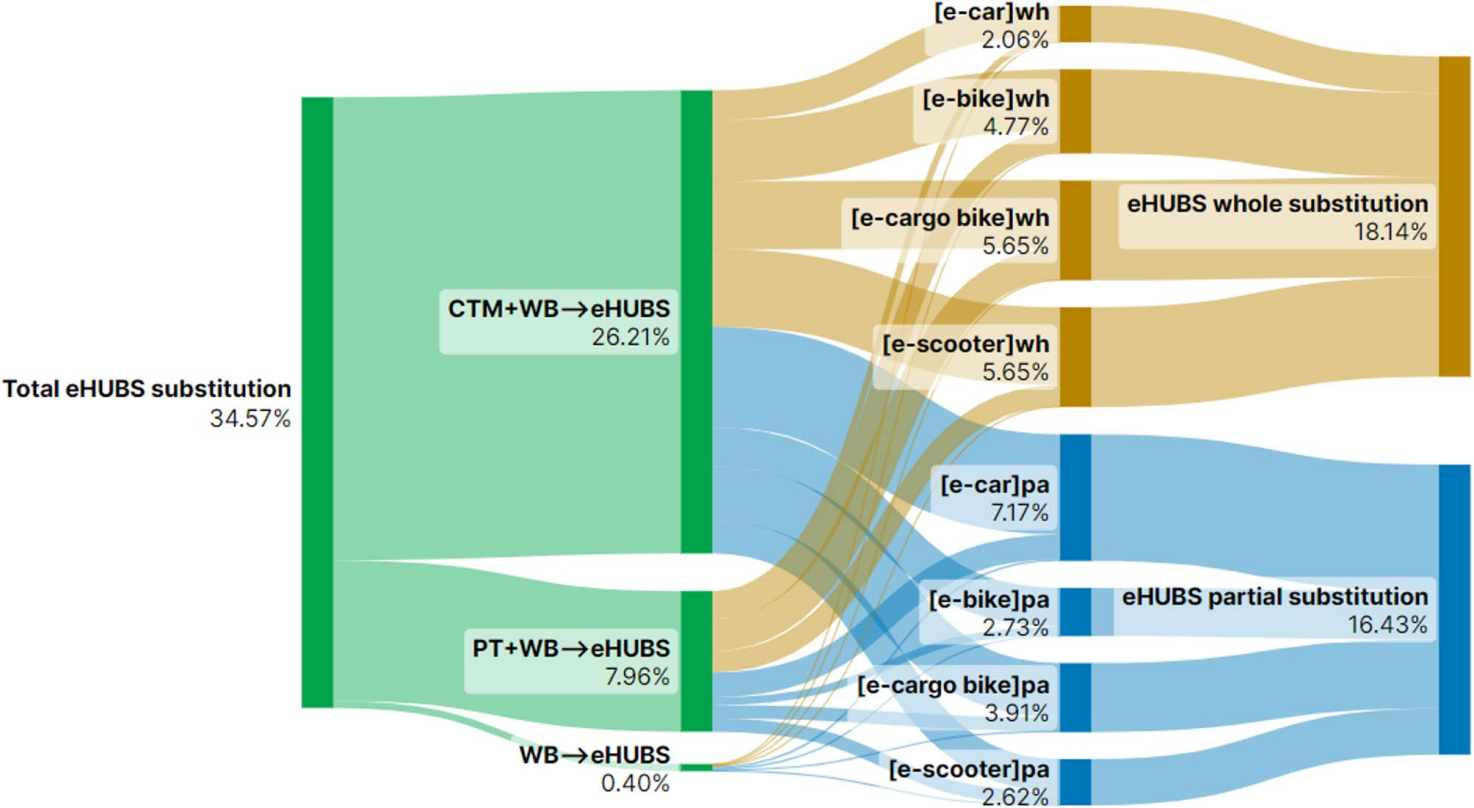
Substitution probabilities within [10, 20 km): whole vs. partial trips. Generated using SankeyArt v1.4 (2025), https://www.sankeyart.com.

**Fig. 9:**
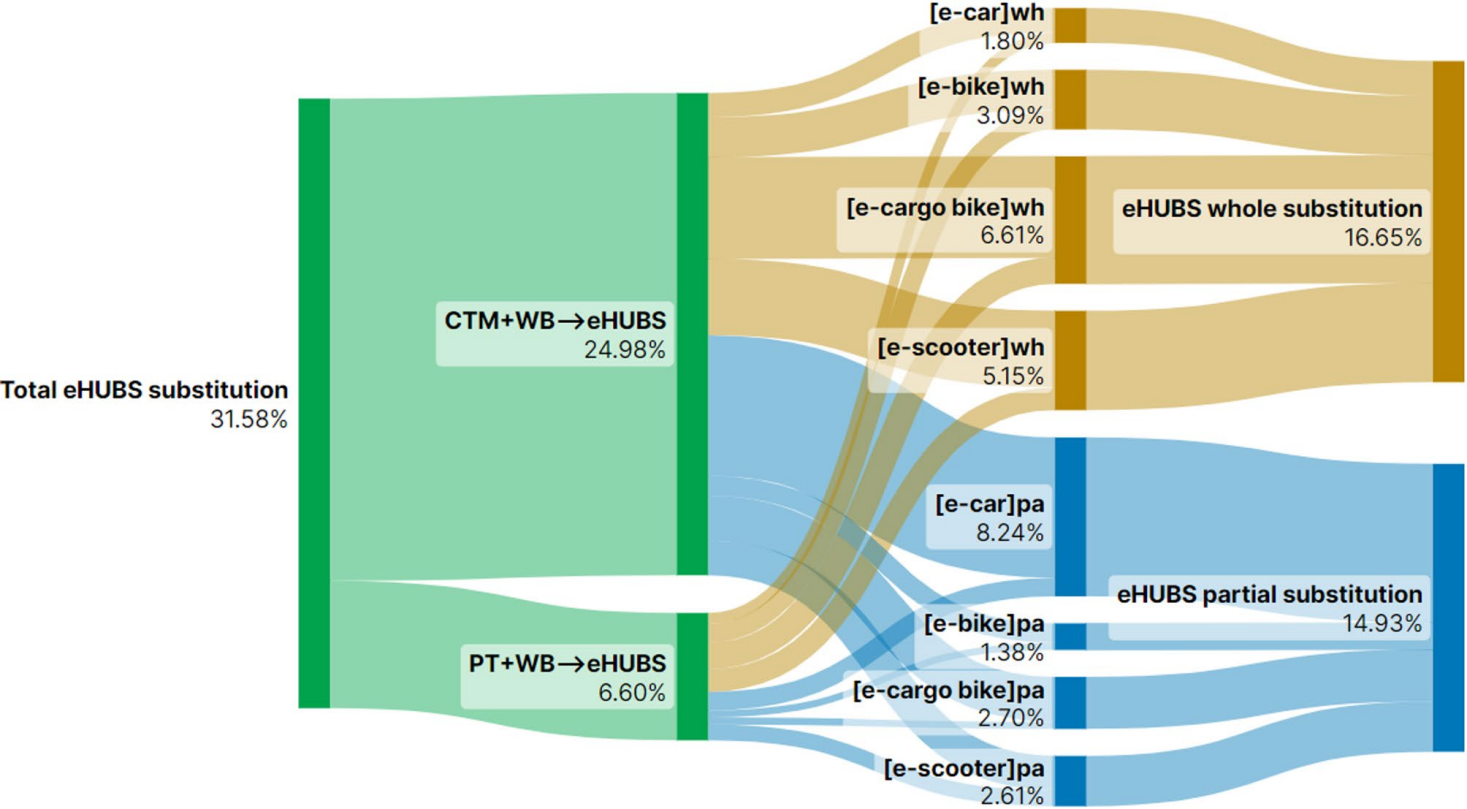
Substitution probabilities within [20 km, $\:+\infty\:$): whole vs. partial trips. Generated using SankeyArt v1.4 (2025), https://www.sankeyart.com.

In the medium-long distance interval [10–20 km), eHUBS integration with public transport reaches its peak at 16.43% (Fig. 8). This rise is due to users shifting from motorised travel (CTM + WB) to a combination of eHUBS and public transport, with e-cars in particular substituting CTM + WB and achieving over 7% (Fig. 8). Combined with the 15.47% of users continuing to use PT + WB (Fig. 4), this brings the overall proportion 8.47% above the pre-eHUBS public transport level (23.43%, Fig. 4), showing eHUBS’s role in promoting longer trips via public transport, with e-cars as a complementary alternative. For long-distance travel (20 km and above), the eHUBS and public transport combination accounts for 14.93% (Fig. 9). Including the 9.30% continuing with PT + WB, the total proportion of public transport users reaches 24.23%, an 8.33% increase from pre-eHUBS levels. Similar to the medium-long interval, more users who previously relied on motorised modes now partially substitute trips with e-cars, completing the remainder with public transport (8.24%). This pattern, particularly relevant for interregional travel, suggests that promoting eHUBS could facilitate a shift from motorised modes to more sustainable public transport options, such as trains and coaches.

Overall, In Greater Manchester, eHUBS achieved a 30%–35% substitution rate, contributing to an 8%–13% increase in public transport use. For trips over 5 km, e-cars notably supported public transport integration, while for shorter distances, e-bikes and e-cargo bikes had limited impact—likely due to concerns over safety and carrying capacity.

In testing robustness, five alternative Likert-scale probability mappings were examined in addition to the baseline (0%, 25%, 50%, 75%, 100%). These scenarios included trimmed extremes, optimistic, conservative, central tendency, and pessimistic variants, each reflecting different assumptions about respondents’ behavioural intent. This design enabled us to test the sensitivity of results under boundary adjustments, more favourable or unfavourable interpretations, and redistributions towards moderation. The findings indicate that, in most scenarios, deviations in overall eHUBS substitution rates are generally contained within $\:\pm\:$1%, while changes for individual modes are typically within $\:\pm\:$0.5% to $\:\pm\:$1.5%. Only under the most pessimistic assumptions do the deviations approach $\:\pm\:$3%. These variations confirm the robustness of the conclusions; details are in Appendix C and results in Tables V–X.

### Quantifying carbon reduction with eHUBS substitution

This section provides a quantitative analysis of the carbon emissions reduction contribution under the eHUBS mode substitution.

#### Calculating equivalent carbon emissions

Firstly, Eq. 7 is used to calculate the travel distance $\:{d}_{ij}$ for each trip within distance interval $\:j$ using conventional travel mode combination $\:i$. In this equation, $\:s$ represents the seven conventional transport modes, $\:\overline{{v}_{s}}$ denotes the average speed (in km/h) of mode $\:s$ in Greater Manchester, and $\:{t}_{s}$ is the time (in hours) a traveller spends using mode $\:s$ during the trip. The travel durations for each mode can be obtained from Section III of the survey. By determining the average speeds of the seven transport modes (see Table 8), the $\:{d}_{ij}$ is calculated.

$$\:{d}_{ij} = {\sum\:}_{s = 1}^{7}\overline{{v}_{s}}{t}_{s}$$7.

**Table 8:** Average speeds of seven travel modes in greater Manchester (Note: speed values are sourced from previous studies conducted in greater Manchester, e.g.^39,46–50^).

Mode	Car	Taxi	Motorbike	Bus	LRT	Bike	Walking
Speed (km/h)	35.6	35.6	48	16	25	10	4.7

Based on Eq. 7, Table 9 presents the total number of trips $\:{N}_{ij}$, the total travel distance $\:{D}_{ij}$, and the average travel distance $\:{\overline{D}}_{ij}$ within distance interval $\:j$ for conventional travel mode combination $\:i$.

**Table 9:** Several key results across 1139 trip records during $\:CO_{2}e$ reduction quantification.

$\:{H}_{j}$	$\:{N}_{j}$	$\:{G}_{i}$	$\:{N}_{ij}$	$\:{D}_{ij}$ (km)	$\:{\overline{D}}_{ij}$ (km)	$\:{Q}_{ij}$ (kg $\:CO_{2}e$)	$\:{q}_{ij}\:(\text{k}\text{g}\:CO_{2}e/\text{p}\text{a}\text{s}\text{s}\text{e}\text{n}\text{g}\text{e}\text{r}/\text{t}\text{r}\text{i}\text{p})$
$\:{H}_{1}$	441	$\:{G}_{1}$	156	477.79	3.06	64.35	0.41
		$\:{G}_{2}$	34	123.26	3.63	10.43	0.31
		$\:{G}_{3}$	251	342.99	1.37	0.00	0.00
$\:{H}_{2}$	296	$\:{G}_{1}$	235	1653.50	7.04	211.05	0.90
		$\:{G}_{2}$	53	411.84	7.77	25.78	0.49
		$\:{G}_{3}$	8	60.32	7.54	0.00	0.00
$\:{H}_{3}$	226	$\:{G}_{1}$	168	2440.28	14.53	325.02	1.93
		$\:{G}_{2}$	53	722.55	13.63	43.69	0.82
		$\:{G}_{3}$	5	70.23	14.05	0.00	0.00
$\:{H}_{4}$	176	$\:{G}_{1}$	147	6530.48	44.43	842.90	5.73
		$\:{G}_{2}$	28	1029.60	36.77	95.27	3.40
		$\:{G}_{3}$	1	21.54	21.54	0.00	0.00

Then, the total carbon emissions equivalent $\:{Q}_{ij}$ (kg $\:CO_{2}e$) for the $\:{N}_{ij}$ trips within distance range $\:j$ using conventional travel mode combination $\:i$ are calculated using Eq. 8.

$$\:{Q}_{ij} = {\sum\:}_{N = 1}^{{N}_{ij}}{\sum\:}_{s = 1}^{7}{C}_{s}\overline{{v}_{s}}{t}_{s}$$8.

The carbon emission factor ($\:{C}_{s}$, kg $\:CO_{2}e$/km) Is a critical parameter for quantifying the carbon intensity of transport mode $\:s$. Table 10 provides the $\:{C}_{s}$ values for various conventional transport modes in the UK, including cars, buses, LRT, electric motorbikes, and taxis^51^. In this study, the $\:{C}_{s}$ values for walking and cycling are assumed to be zero, excluding the carbon footprint of food consumed for energy. While food-related emissions can be associated with active travel modes, they are excluded here to maintain focus on transport-sector carbon reduction. This allows for a clearer analysis of whether shared electric mobility (eHUBS) displaces zero-emission modes that are central to sustainable urban transport, without introducing additional complexity that may obscure substitution effects. Additionally, the $\:{C}_{s}$ values for buses, LRT, and taxis are expressed in kg$\:CO_{2}e$/passenger/km. For private car journeys with multiple passengers, the vehicle’s total carbon emission factor is divided by 1.59 to estimate per-passenger emissions, based on the average occupancy rate for private cars in the UK^52^. Table 9 presents the $\:{Q}_{ij}$ values derived from Eq. 8, facilitating the calculation of the average $\:CO_{2}e$ emissions per person ($\:{q}_{ij}$), also detailed in Table 9, for trips conducted using mode combination $\:i$ within distance interval $\:j$.

**Table 10:** The summary of carbon emission factor $\:{C}_{s}$ for UK conventional transport modes^51^.

Modes		$\:{C}_{s}\:(\mathbf{k}\mathbf{g}\:{\varvec{C}\varvec{O}}_{{2}^{\varvec{e}}}/\mathbf{k}\mathbf{m})$			
Bus		0.10778/passenger			
LRT		0.02861/passenger			
Motorbike		0.1009			

	Segment	Diesel	Petrol	PHEV	Electric
Car	A: Mini	0.108294	0.13421	×	0.04045
	B: Small	0.132764	0.14802	0.05255	0.04476
	C: Medium	0.144614	0.17162	0.08342	0.04878
	D: Large	0.161974	0.19923	0.0882	0.04797
	E: Executive	0.174684	0.21999	0.08975	0.04646
	F: Luxury	0.21243414	0.32708	0.0920	0.05933
	S: Sports	0.170414	0.24145	0.09359	0.07820
	M: Multi-purpose	0.202964	0.21060	0.10371	0.06018
	J: Sport utility	0.177844	0.19118	0.09035	0.06881

Subsequently, based on the results of $\:P\left({E}_{k}{G}_{i}|{H}_{j}\right)$ and $\:P\left({F}_{k}{E}_{k}{G}_{i}|{H}_{j}\right)$ from Eqs. 3 and 5, Eqs. 9 and 10 are employed to calculate the number of trips $\:{T}_{kij}^{wh}$ and $\:{T}_{kij}^{pa}$ within distance interval $\:j$, where conventional travel mode combination $\:i$ transitions to the four eHUBS modes $\:k$ under whole-trip and partial-trip substitution scenarios, respectively. The specific results are presented in Appendix D (Table XI).

9 $$\:{T}_{kij}^{wh} = {N}_{ij}*\left[P\left({E}_{k}{G}_{i}|{H}_{j}\right)-P\left({F}_{k}{E}_{k}{G}_{i}|{H}_{j}\right)\right]$$

10 $$\:{T}_{kij}^{pa}\: = {N}_{ij}*P\left({F}_{k}{E}_{k}{G}_{i}|{H}_{j}\right)$$

The $\:CO_{2}e$ generated by eHUBS following the whole-trip and partial-trip substitution of each conventional travel mode combination $\:i\:$across distance intervals $\:j\:$are now calculated. In the scenario where the mode combination $\:i$ are entirely substituted by eHUBS mode $\:k$ within travel interval $\:j$, the $\:CO_{2}e$ produced by eHUBS mode $\:k$, $\:{M}_{kij}^{wh}$, are decided using Eq. 11.

11 $$\:{M}_{kij}^{wh} = {T}_{kij}^{wh}*{l}_{kij}^{wh}*\overline{D}*{C}_{k}$$

The term $\:{l}_{kij}^{wh}$ denotes the distance conversion factor within the travel interval $\:j$. It quantifies the conversion of the travel distance associated with conventional travel mode combination $\:i$ into the corresponding distance when eHUBS mode $\:k$ fully substitutes the combination $\:i$ for the same trip, assuming identical trip origin and destination points. The average travel distance $\:\overline{D}$ from Table 9 is used as the typical travel distance for each travel group. $\:{C}_{k}\:$represents the carbon emission factor for the four eHUBS modes. Based on the car market segments defined by the European Commission^45^ and the carbon emission factors established by the UK government for different vehicle types^51^, this paper selects the $\:{C}_{k}$ ($\:k$ = $\:1$, 0.04476 $\:CO_{2}e$ kg/km, refer to Table 10) designated for B-segment electric cars to represent the carbon emission factor for e-cars within eHUBS. This choice is informed by the typically smaller dimensions of the electric cars used in eHUBS. For the other three eHUBS modes (i.e., $\:k =$1, 2, 3), $\:{C}_{k}$ are determined based on Eq. 12.

12 $$\:{C}_{k}\left(\text{k}\text{g}\frac{{CO}_{{2}^{e}}}{\text{k}\text{m}}\right) = \text{C}\text{a}\text{r}\text{b}\text{o}\text{n}\:\text{i}\text{n}\text{t}\text{e}\text{n}\text{s}\text{i}\text{t}\text{y}\:\left(\text{k}\text{g}\frac{{CO}_{2}e}{\text{k}\text{W}\text{h}}\right)\text{*}\text{E}\text{n}\text{e}\text{r}\text{g}\text{y}\:\text{c}\text{o}\text{n}\text{s}\text{u}\text{m}\text{p}\text{t}\text{i}\text{o}\text{n}\:\text{o}\text{f}\:k\left(\frac{\text{k}\text{W}\text{h}}{\text{k}\text{m}}\right)\:$$

Where carbon intensity refers to the amount of $\:{CO}_{2}e$ produced per kilowatt-hour (kWh) of electricity generated by mixed power plants. In the UK, this value is 0.1620 kg $\:{CO}_{2}e$/kWh in 2023^53^. The energy consumption of e-bikes, e-cargo bikes, and e-scooters is 0.0200 kWh/km^54^, 0.0170 kWh/km^55^, and 0.0474 kWh/km^56^, respectively. Then, according to Eq. 12, the carbon emission factors for e-bikes, e-cargo bikes, and e-scooters are 0.00324 kg $\:{CO}_{2}e$/km, 0.00275 kg $\:{CO}_{2}e$/km, and 0.00767 kg $\:{CO}_{2}e$/km, respectively. Therefore, $\:{M}_{kij}^{wh}$ can be determined according to Eqs. 11 and 12, which are shown in Table 11.

Next, $\:{CO}_{2}e$ arising from the partial-trip substitution of conventional travel mode combination $\:i$ with eHUBS mode$\:\:k$ within distance interval $\:j$, $\:{M}_{kij}^{pa}$, is calculated. Based on Eq. 13, these emissions include two components: the first is the emissions generated by the partial replacement of the conventional travel mode combination$\:\:i$ with eHUBS, and the second is the emissions produced by completing the remaining journey using public transport.

13 $$\:{M}_{kij}^{pa} = {T}_{kij}^{pa}{l}_{kij}^{pa}\overline{D}[{r}_{j}{C}_{k}+(1-{r}_{j}\left){C}_{PT}\right]$$

Where $\:{l}_{kij}^{pa}$ means the distance conversion factor when partially substituting conventional travel mode combination $\:i$ with eHUBS mode $\:k$ and finish it by using public transport. The carbon emission factor ($\:{C}_{s}$) for buses and LRT are 0.10778 kg $\:CO_{2}e$/km and 0.02861 kg$\:CO_{2}e$/km, respectively. In this study, the average value of 0.0682 kg $\:CO_{2}e$/km is utilised to represent $\:{C}_{PT}$. Moreover, $\:{r}_{j}\:(0\le\:{r}_{j}\le\:1)$ is a proportion factor representing the proportion of the WB distance relative to the total PT+WB travel distance within distance interval $\:j$. The reason for introducing $\:{r}_{j}$ is that, in partial substitution scenario, eHUBS is assumed to primarily substitute the portion of the trip that connects the traveller from their starting location to the public transport hub. In this paper, $\:{r}_{j}$ is obtained by analysing 168 PT + WB records, yielding the following values for the four travel intervals $\:j$: $\:{r}_{1}$ = $\:{r}_{2}$ = 0.19, $\:{r}_{3}$ = 0.16, and $\:{r}_{4}$ = 0.18. Overall, in Eq. 13, $\:{r}_{j}{C}_{k}$ represents the unit $\:{CO}_{2}e$ for the portion of the trip where eHUBS mode $\:k$ partially replaces the original trip, while $\:(1-{r}_{j}){C}_{PT}$ represents the unit $\:{CO}_{2}e$ for the remainder of the trip completed using public transport. All results of $\:{M}_{kij}^{pa}$ are concluded in Table 11.

**Table 11:** $\:{CO}_{2}e$ Emissions from whole-trip ($\:{M}_{kij}^{wh}$) and partial-trip ($\:{M}_{kij}^{pa}$) eHUBS substitution of conventional modes across four distances interval $\:j$.

$\:{H}_{j}$	$\:{G}_{i}$	$\:{M}_{kij}^{wh}$ & $\:{M}_{kij}^{pa}$	$\:k = 1$	$\:k = 2$	$\:k = 3$	$\:k = 4$
$\:{H}_{1}$	$\:{G}_{1}$	$\:{M}_{k11}^{wh}$	0.41$\:{l}_{111}^{wh}$	0.02$\:{l}_{211}^{wh}$	0.02$\:{l}_{311}^{wh}$	0.02$\:{l}_{411}^{wh}$
		$\:{M}_{k11}^{pa}$	0.39$\:{l}_{111}^{pa}$	0.75$\:{l}_{211}^{pa}$	0.17$\:{l}_{311}^{pa}$	0.52$\:{l}_{411}^{pa}$
	$\:{G}_{2}$	$\:{M}_{k21}^{wh}$	0.00	0.00	0.00	0.00
		$\:{M}_{k21}^{pa}$	0.00	0.00	0.00	0.00
	$\:{G}_{3}$	$\:{M}_{k31}^{wh}$	0.18$\:{l}_{131}^{wh}$	0.03$\:{l}_{231}^{wh}$	0.03$\:{l}_{331}^{wh}$	0.04$\:{l}_{431}^{pa}$
		$\:{M}_{k31}^{pa}$	0.09$\:{l}_{131}^{pa}$	0.69$\:{l}_{231}^{pa}$	0.31$\:{l}_{331}^{pa}$	0.54$\:{l}_{431}^{pa}$
$\:{H}_{2}$	$\:{G}_{1}$	$\:{M}_{k12}^{wh}$	4.10$\:{l}_{112}^{wh}$	0.14$\:{l}_{212}^{wh}$	0.12$\:{l}_{312}^{wh}$	0.27$\:{l}_{412}^{wh}$
		$\:{M}_{k12}^{pa}$	2.24$\:{l}_{112}^{pa}$	3.93$\:{l}_{212}^{pa}$	1.96$\:{l}_{312}^{pa}$	3.19$\:{l}_{412}^{pa}$
	$\:{G}_{2}$	$\:{M}_{k22}^{wh}$	0.00	0.00	0.00	0.00
		$\:{M}_{k22}^{pa}$	0.00	0.43$\:{l}_{222}^{pa}$	0.43$\:{l}_{322}^{pa}$	0.00
	$\:{G}_{3}$	$\:{M}_{k32}^{wh}$	0.00	0.00	0.00	0.00
		$\:{M}_{k32}^{pa}$	0.00	0.00	0.00	0.00
$\:{H}_{3}$	$\:{G}_{1}$	$\:{M}_{k13}^{wh}$	6.50$\:{l}_{113}^{wh}$	0.19$\:{l}_{213}^{wh}$	0.20$\:{l}_{313}^{wh}$	0.33$\:{l}_{413}^{wh}$
		$\:{M}_{k13}^{pa}$	2.81$\:{l}_{113}^{pa}$	5.04$\:{l}_{213}^{pa}$	5.03$\:{l}_{313}^{pa}$	5.95$\:{l}_{413}^{pa}$
	$\:{G}_{2}$	$\:{M}_{k23}^{wh}$	0.61$\:{l}_{123}^{wh}$	0.00	0.00	0.00
		$\:{M}_{k23}^{pa}$	0.00	0.79$\:{l}_{223}^{pa}$	0.79$\:{l}_{323}^{pa}$	0.80$\:{l}_{423}^{pa}$
	$\:{G}_{3}$	$\:{M}_{k33}^{wh}$	0.00	0.00	0.00	0.00
		$\:{M}_{k33}^{pa}$	0.00	0.00	0.00	0.00
$\:{H}_{4}$	$\:{G}_{1}$	$\:{M}_{k14}^{wh}$	21.88$\:{l}_{114}^{wh}$	0.14$\:{l}_{214}^{wh}$	0.37$\:{l}_{314}^{wh}$	1.02$\:{l}_{414}^{wh}$
		$\:{M}_{k14}^{pa}$	5.69$\:{l}_{114}^{pa}$	7.53$\:{l}_{214}^{pa}$	20.05$\:{l}_{314}^{pa}$	15.28$\:{l}_{414}^{pa}$
	$\:{G}_{2}$	$\:{M}_{k24}^{wh}$	0.00	0.00	0.00	0.00
		$\:{M}_{k24}^{pa}$	0.00	0.00	0.00	0.00
	$\:{G}_{3}$	$\:{M}_{k34}^{wh}$	0.00	0.00	0.00	0.00
		$\:{M}_{k34}^{pa}$	0.00	0.00	0.00	0.00

The distance conversion factor $\:{l}_{kij}$ is now determined for both scenarios. It is assumed that $\:{l}_{11j}^{wh} = {l}_{11j}^{pa} = 1$, as the distance is considered equal when substituting motorised vehicle ($\:i$ = 1) with e-car ($\:k$ = 1). Also, It has been suggested that public transport trips are generally 1.4 to 2.6 times longer than e-car trips^57^, thus $\:{l}_{12j}^{wh}$ = $\:{l}_{12j}^{pa}$ = 0.5. Moreover, it is posited that, for the same origin and destination, the distance covered by e-car is greater than that covered by WB, leading to the assignment of $\:{l}_{13j}^{wh}$ = $\:{l}_{13j}^{pa}$ = 1.3. Additionally, the assumption is made that for the same trip, the distance travelled using e-bikes, e-cargo bikes, and e-scooters is shorter than that generated by both CTM+WB and PT+WB. Hence, the values $\:{l}_{21j}^{wh} = {l}_{21j}^{pa} = {l}_{22j}^{wh} = {l}_{22j}^{wh} = {l}_{31j}^{pa} = {l}_{31j}^{wh} = {l}_{32j}^{wh} = {l}_{32j}^{pa} = {l}_{41j}^{wh} = {l}_{41j}^{pa} = {l}_{42j}^{wh} = {l}_{42j}^{pa}$ = 0.8 are applied. Finally, we assume that the distance covered by e-bikes, e-cargo bikes, and e-scooters is equal to that of WB for the same trip. Thus, $\:{l}_{23j}^{wh} = {l}_{23j}^{pa} = {l}_{33j}^{wh} = {l}_{33j}^{pa} = {l}_{43j}^{wh} = {l}_{43j}^{pa} = 1$ is set.

Based on these assumptions, the changes in $\:{CO}_{2}e$ for current trips and potentially after the implementation of eHUBS $\:\varDelta\:{Q}_{ij}$ can be calculated using Eq. 14. Specifically, $\:{Q}_{ij}$ represents the total $\:CO_{2}e$ produced by conventional travel mode combination $\:i$ within travel distance interval$\:\:j$ prior to the introduction of eHUBS. $\:\left({N}_{ij}-{\sum\:}_{k = 1}^{4}{T}_{kij}^{wh}-{\sum\:}_{k = 1}^{4}{T}_{kij}^{pa}\right){q}_{ij}$ signifies the $\:CO_{2}e$ generated by the continued use of conventional travel mode combination $\:i$ even after the implementation of eHUBS in Greater Manchester. Additionally, $\:{\sum\:}_{k = 1}^{4}{M}_{kij}^{wh}+{\sum\:}_{k = 1}^{4}{M}_{kij}^{pa}$ quantifies the $\:CO_{2}e$ that were previously attributed to the use of mode combination $\:i$, now redirected to the emissions associated with employing eHUBS following its introduction.

14 $$\:\varDelta\:{Q}_{ij} = {Q}_{ij}-\left({N}_{ij}-{\sum\:}_{k = 1}^{4}{T}_{kij}^{wh}-{\sum\:}_{k = 1}^{4}{T}_{kij}^{pa}\right){q}_{ij}-\left({\sum\:}_{k = 1}^{4}{M}_{kij}^{wh}+{\sum\:}_{k = 1}^{4}{M}_{kij}^{pa}\right)$$

**Fig. 10:**
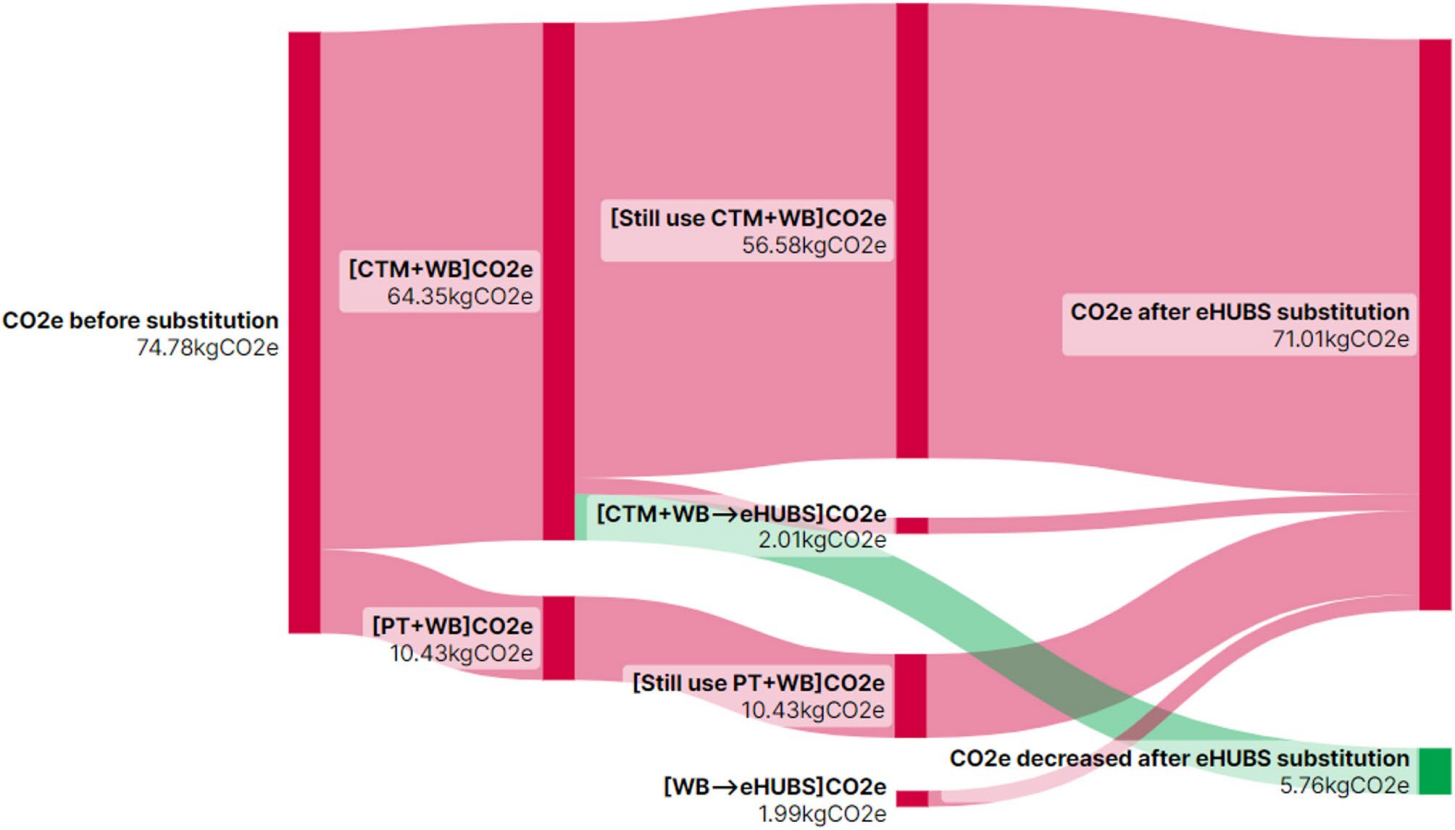
$\:{CO}_{2}e$ reduction ($\:\varDelta\:{Q}_{i1}$) from eHUBS substitution within [0, 5 km). Generated using SankeyArt v1.4 (2025), https://www.sankeyart.com.

**Fig. 11:**
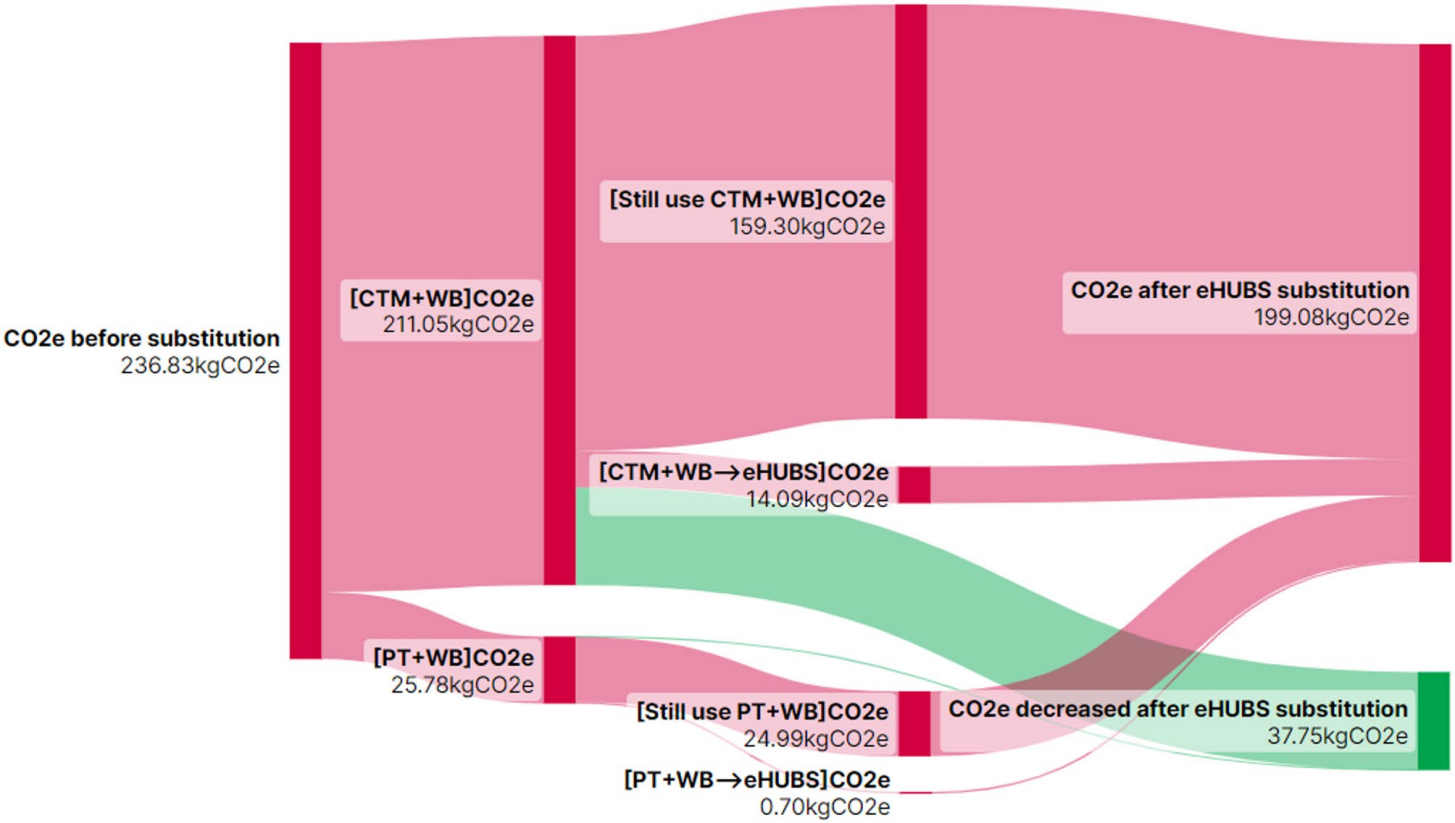
$\:{CO}_{2}e$ reduction ($\:\varDelta\:{Q}_{i2}$) from eHUBS substitution within [5, 10 km). Generated using SankeyArt v1.4 (2025), https://www.sankeyart.com.

**Fig. 12:**
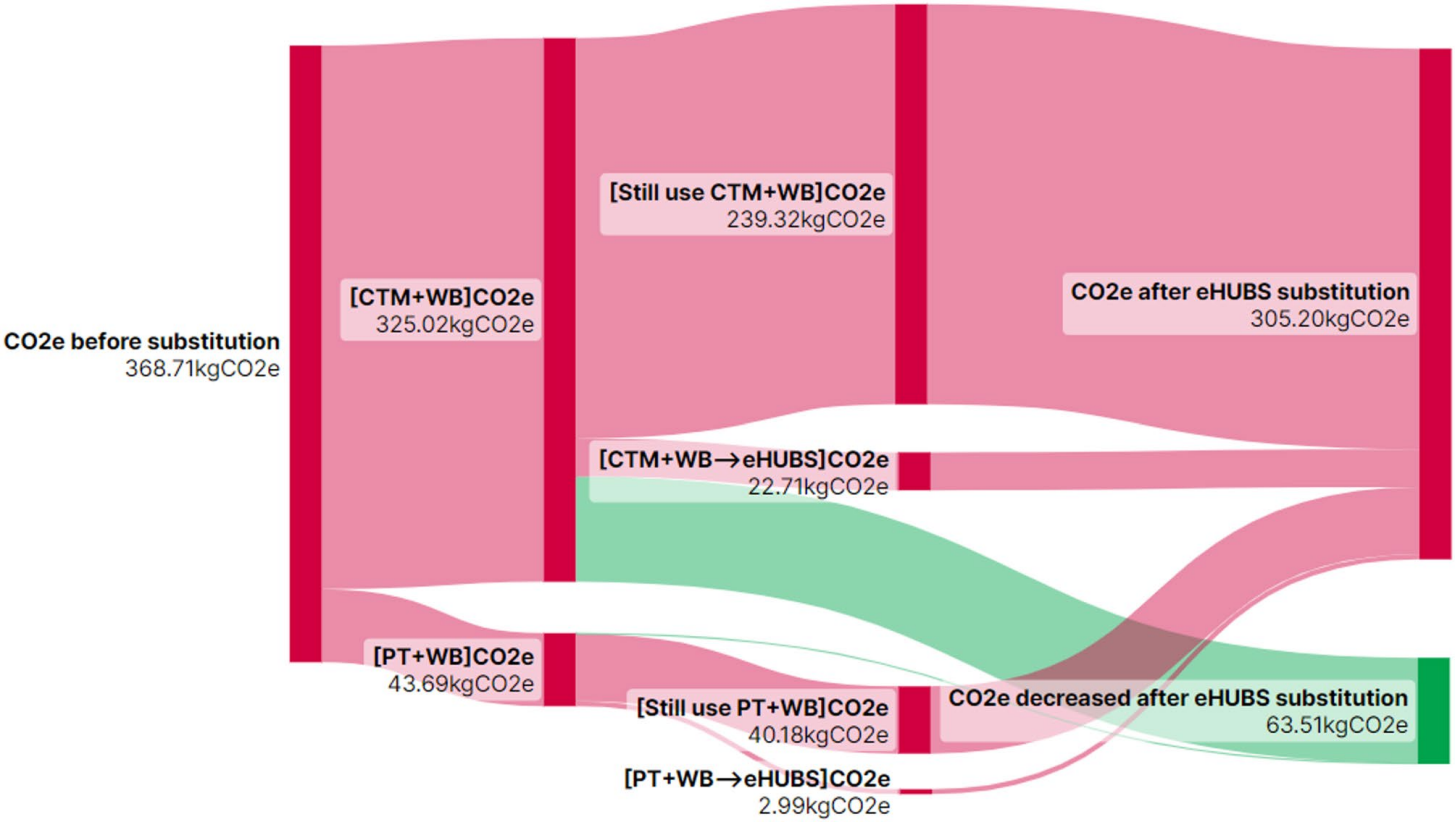
$\:{CO}_{2}e$ reduction ($\:\varDelta\:{Q}_{i3}$) from eHUBS substitution within [10, 20 km). Generated using SankeyArt v1.4 (2025), https://www.sankeyart.com.

**Fig. 13:**
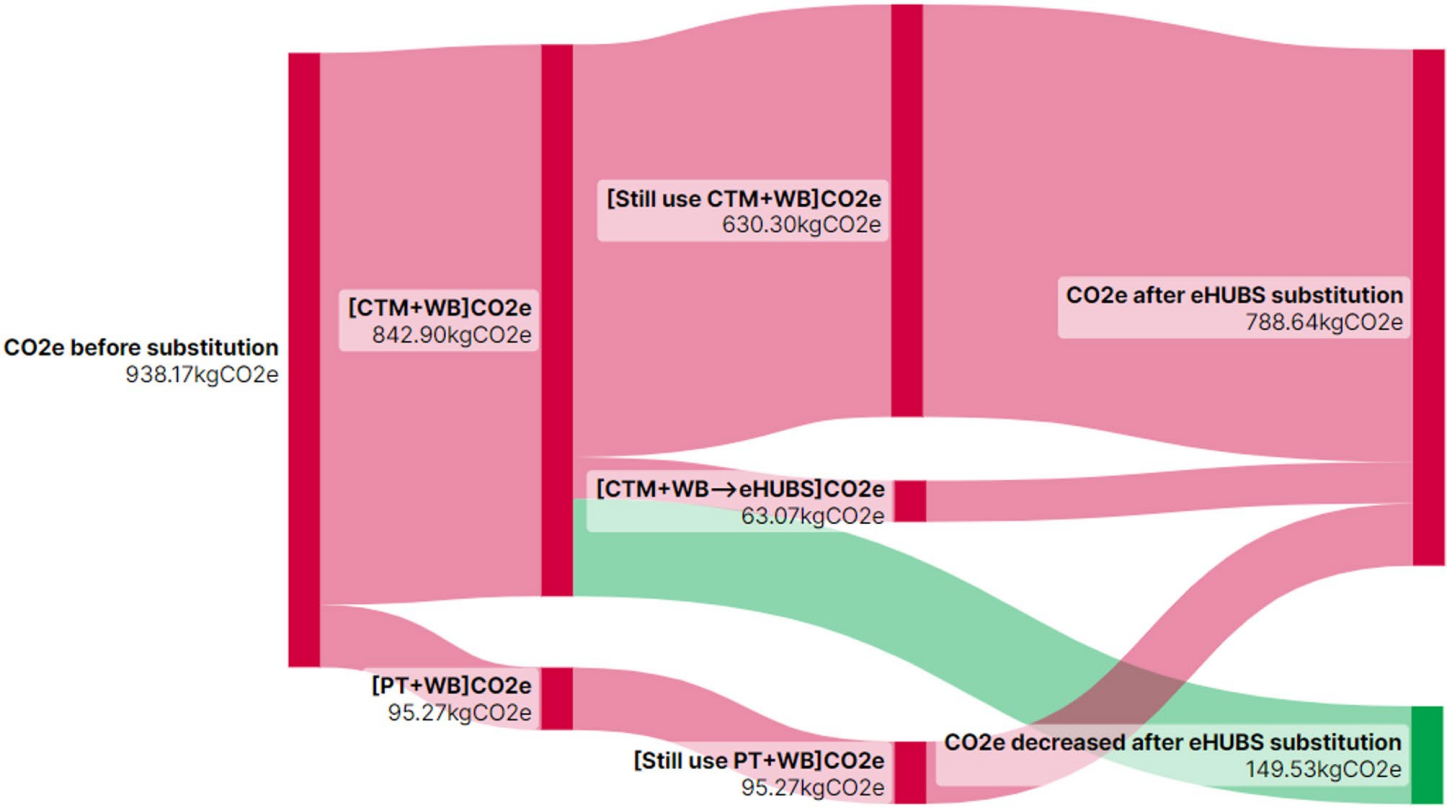
$\:{CO}_{2}e$ reduction ($\:\varDelta\:{Q}_{i4}$) from eHUBS substitution within [20 km,+$\:\infty\:$). Generated using SankeyArt v1.4 (2025), https://www.sankeyart.com.

The changes in $\:{CO}_{2}e$ $\:(\varDelta\:{Q}_{ij})$ from substituting conventional travel mode combination $\:i$ with eHUBS across distance interval $\:j$ are illustrated in Figs. 10, 11, 12 and 13 using Sankey diagrams. Note that the distance conversion factor may vary due to factors like weather, road gradients, and complexity. While relevant, these factors are outside this paper’s scope but may be considered in detail in future research.

**Fig. 14:**
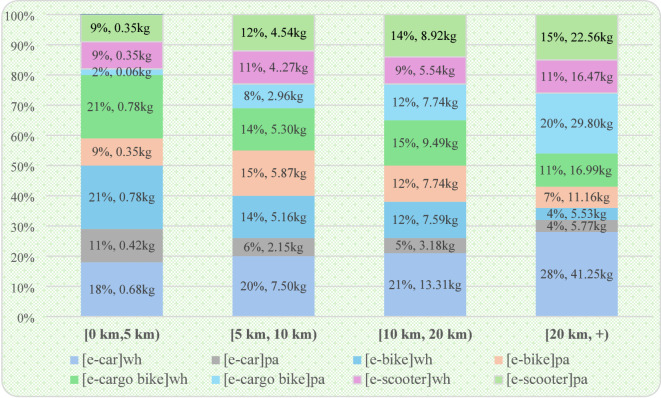
Contribution of the 4 eHUBS modes to $\:{CO}_{2}e$ reduction ($\:{\varDelta\:M}_{kj}^{wh}$ and $\:{\varDelta\:M}_{kj}^{pa}$) across 4 travel intervals for whole and partial-trip scenarios. Generated using Microsoft Excel.

To assess each eHUBS mode’s contribution to $\:CO_{2}e$ reduction within travel interval $\:j$ for whole-trip and partial-trip substitution, we define $\:{\varDelta\:M}_{kij}^{wh}$ and $\:{\varDelta\:M}_{kij}^{pa}$ (Eqs. 15 and 16). Moreover, to visually illustrate the contribution of the four eHUBS modes to $\:CO_{2}e$ reduction under both scenarios, Fig. 14 is created.

15 $$\Delta M_{{kj}}^{{wh}} = \mathop \sum \limits_{{i = 1}}^{3} \left( {T_{{kij}}^{{wh}} q_{{ij}} - M_{{kij}}^{{wh}} } \right)$$

16 $$\Delta M_{{kj}}^{{pa}} = \mathop \sum \limits_{{i = 1}}^{3} \left( {T_{{kij}}^{{pa}} q_{{ij}} - M_{{kij}}^{{pa}} } \right)$$

### Analysis of $\:\varvec{C}\varvec{O}_{2}\varvec{e}$ reduction under eHUBS substitution

Figures 10, 11, 12 and 13 demonstrate that the implementation of eHUBS in Greater Manchester reduces $\:CO_{2}e$ emissions across four distinct travel distance intervals. Specifically, total emissions decreased from 74.78 kg, 236.83 kg, 368.71 kg, and 938.17 kg $\:CO_{2}e$ to 71.01 kg, 199.08 kg, 305.20 kg, and 788.64 kg $\:CO_{2}e$, respectively, corresponding to reductions of 5.04%, 15.94%, 17.22%, and 15.99%. These findings indicate that, with the exception of short-distance trips, the substitution effect of eHUBS makes a substantial contribution to carbon mitigation.

According to Fig. 10, in the short-distance travel interval [0 km, 5 km), although total emissions fell to 71.01 kg, 42 trips that were originally made by walking or cycling were replaced by eHUBS modes, resulting in an additional 1.99 kg $\:CO_{2}e$ emissions. This suggests that the introduction of eHUBS in short-distance scenarios may undermine the emission benefits typically associated with zero-carbon modes such as walking and cycling. Nonetheless, the same figure also shows that the substitution of motorised modes with eHUBS led to a total reduction of 5.76 kg $\:CO_{2}e$, which is nearly three times the emissions increase caused by replacing zero-carbon trips. Therefore, even within short-distance intervals, eHUBS maintain a notable net carbon reduction effect. Figures 11, 12 and 13 further illustrate that, across medium-short, medium-long, and long-distance travel intervals, the substitution of conventional modes by eHUBS leads to $\:CO_{2}e$ reductions of approximately 15% to 18%. These reductions are primarily attributable to the replacement of private motorised transport, with minimal impact on public transit or active travel. As the travel distance increases, the total volume of emissions reduction grows significantly, from 37.75 kg to 149.53 kg $\:CO_{2}e$.

Additionally, Fig. 14 presents the proportional $\:CO_{2}e$ reductions for each of the four eHUBS modes under “whole substitution” and “partial substitution” scenarios within specific travel distance intervals. For instance, in the [0, 5) km short-distance segment, the emission contributions of e-car, e-bike, and e-cargo bike were 29% (1.10 kg $\:CO_{2}e$), 30% (1.13 kg $\:CO_{2}e$), and 23% (0.84 kg $\:CO_{2}e$), respectively. At first glance, the emission reduction from e-car appears comparable to or even greater than that of e-bike, which may seem counterintuitive. However, this outcome is largely a manifestation of the carbon rebound effect. As shown in the Sankey diagram (Fig. 2), although e-car accounts for 4.91% of all substitutions, two-thirds of which effectively replace motorised combinations (CTM + WB), approximately one-third replaces walking or cycling, both of which are zero-carbon modes. According to Table 10, the emission factor of e-cars ranges from 0.04 to 0.05 kg $\:CO_{2}e$/km (depending on vehicle segment A–E), significantly higher than that of e-bikes (0.00324 kg $\:CO_{2}e$/km) and e-cargo bikes (0.00275 kg $\:CO_{2}e$/km). Consequently, while e-cars do substitute higher-emission trips, their partial replacement of zero-carbon trips offsets much of their net benefit, explaining why e-cars underperform in terms of short-distance emission savings compared to e-bikes and only slightly outperform e-cargo bikes. This carbon rebound effect is mitigated in longer travel distances. In the 20 km + interval, e-cars contribute 47 kg $\:CO_{2}e$ reduction (32% of the total), surpassing both e-bike (16.69 kg $\:CO_{2}e$, 11%) and e-cargo bike (46.76 kg $\:CO_{2}e$, 31%). However, below 20 km, e-cars remain less competitive. In the [5, 10 km) range, e-car achieves 9.65 kg $\:CO_{2}e$ reduction (26%), lower than e-bike (11.03 kg, 29%) and slightly above e-cargo bike (8.26 kg, 22%). In the [10, 20 km) range, the proportions are 26% (e-car), 24% (e-bike), and 27% (e-cargo bike), indicating that e-car does not exhibit a consistent advantage in emission reductions.

This pattern is further explained by the underlying substitution structure between full and partial replacement. Figures 6, 7, 8 and 9 reveal that e-cars are more frequently adopted as a partial substitution mode across all distance intervals. For example, in the [0, 5 km) segment, the share of full substitution is 1.46% compared to 3.45% for partial substitution. In the [10, 20 km) interval, the respective figures are 2.06% and 7.17%, and in the 20 km + category, they reach 1.80% and 8.24%. This suggests that most e-car users deploy them to bridge the first-mile gap between origin and public transit (such as LRT or buses) rather than as a full-trip replacement. However, the emission factor of public transport (e.g., buses at 0.10778 kg $\:CO_{2}e$/km) is notably higher than that of e-cars (~ 0.04 kg $\:CO_{2}e$/km), thereby diluting the emission benefits when these two modes are combined. As such, the actual reduction associated with e-car usage, while not negligible, falls short of its full potential.

In contrast, Fig. 6 indicates that e-bikes and e-cargo bikes are more employed as full substitutes for short-distance travel ([0, 5 km)), highlighting their greater potential for independent replacement. This trend is reflected in the emission data: as shown in Fig. 14, in the [0, 5 km) interval, e-bike under full substitution achieves 0.78 kg $\:CO_{2}e$ reduction (21% of total), higher than the partial substitution effect of 0.35 kg $\:CO_{2}e$ (9%). Similarly, e-cargo bike achieves 0.78 kg $\:CO_{2}e$ under full substitution (21%), compared to only 0.06 kg $\:CO_{2}e$ (2%) under partial substitution. However, this trend reverses in longer distance intervals. For trips over 20 km, the full substitution reduction for e-bike is 5.53 kg $\:CO_{2}e$ (4%), while partial substitution accounts for 11.16 kg $\:CO_{2}e$ (7%). The effect is more pronounced for e-cargo bikes, with full substitution yielding 16.99 kg $\:CO_{2}e$ (11%) and partial substitution achieving a higher 29.8% (46.76 kg $\:CO_{2}e$). Interestingly, these outcomes do not align with the substitution rates in Fig. 9, which show that for distances over 20 km, the full substitution rates for e-bike and e-cargo bike are 3.09% and 6.61%, respectively, higher than their partial substitution rates (1.38% and 2.7%). This discrepancy suggests that users prefer full substitution over longer distances. The simultaneous presence of low partial substitution rates and high emission reductions implies that such substitutions predominantly replace motorised modes (e.g., CTM + WB), especially for first-mile segments to transport hubs, thus yielding high carbon savings. Additionally, Fig. 14 reveals that e-scooters exhibit a clear trend of increasing emission reduction with travel distance: from 0.35 kg $\:CO_{2}e$ (9%) in [0, 5) km to 22.56 kg $\:CO_{2}e$ (22.56%) in the 20 km + interval. This indicates that e-scooters are particularly effective in reducing emissions in long-distance trips that involve intermodal connections or commercial travel needs.

Overall, apart from short-distance travel, eHUBS demonstrate robust carbon reduction performance across all distance intervals. Overall, by substituting 30%–35% of conventional travel modes, eHUBS contribute to total $\:CO_{2}e$ reductions of 15%–18% in medium to long-distance travel. These benefits stem from replacing high-emission modes such as private cars, rather than public transit or active travel. This finding aligns with prior literature on the carbon mitigation potential of shared electric and cargo bicycles (e.g., references [23], [26]). While e-bikes and e-cargo bikes are more suited for full substitution in short-distance scenarios, with stronger emission reduction effects under this mode, the emission-saving effectiveness of e-scooters is more dependent on their partial substitution roles in longer-distance journeys.

## Discussion and conclusion

This study assesses the substitution effects of shared electric mobility hubs (eHUBS) in Greater Manchester, drawing on travel and usage data from 1,139 respondents. The research aims to evaluate the effectiveness of eHUBS in replacing conventional travel mode combinations, including motor vehicle use, public transport, and zero-carbon options such as walking and cycling. The research focuses on eHUBS substitution effects and their integration with public transport. Additionally, this study quantifies the carbon reduction achieved through potential eHUBS trip substitution. The findings indicate that eHUBS shared services replace conventional travel modes across various travel distance intervals, demonstrating significant potential to enhance public transport usage and reduce carbon emissions. This study presents the following key findings:

1. eHUBS mode substitution shows distinct patterns across travel distance intervals, with rates consistently between 30% and 35% in all four intervals. In short-distance travel [0 km, 5 km), the 31.17% substitution rate primarily replaces zero-carbon modes like walking and cycling, significantly increasing electric bike use. For medium-short trips [5 km, 10 km), the substitution rate slightly drops to 30.57%, but the shift from motorised travel modes becomes more prominent. At medium-long distances [10 km, 20 km), the rate rises to 34.57%, focusing on replacing motorised travel (26.21%) and impacting public transport modes, with travellers preferring higher-performance electric vehicles such as e-cargo bikes and electric cars. For long-distance travel (20 km and beyond), the substitution rate stabilises at 31.58%, with electric cars as the main replacement. E-cargo bikes also maintain a strong substitution rate in long-distance travel, likely linked to shopping trips.
2. This study indicates that introducing eHUBS, with a substitution rate of 30%-35%, can increase public transport usage in Greater Manchester by 8%-13%, effectively promoting public transport usage across various travel distance intervals. Specifically, in short-distance trips ($\:<$5 km), e-bikes and e-cargo bikes serve as valuable connectors to public transport, significantly enhancing its usage. For distances over 5 km, the combination of e-cars with public transport is particularly effective in substituting traditional motor vehicle travel, helping shift some motor vehicle-dependent travellers towards e-car and public transport combinations. However, e-scooters showed limited effectiveness in promoting public transport use, possibly due to concerns about their safety and load capacity. Although the substitution rate of eHUBS for public transport slightly decreases as travel distance increases, public transport’s advantages in long-distance travel, such as reducing urban congestion and carbon footprint, highlight the continued role of eHUBS in promoting sustainable urban mobility in Greater Manchester.
3. eHUBS demonstrates significant carbon reduction effects across various travel distance intervals, with these benefits becoming more pronounced as travel distance increases. Data analysis reveals that for short-distance trips (0–5 km), eHUBS reduces emissions by approximately 5% of the original output, while for trips exceeding 5 km, this reduction rate rises to 15%-18%. This trend is primarily due to the effective replacement of traditional motor vehicle use across different distance ranges. In scenarios where eHUBS fully replaces conventional modes, electric cars show increasing contributions to carbon reduction as distance grows. In partial substitutions, e-cargo bikes and e-scooters exhibit notable carbon reduction potential, particularly in medium-long and long-distance intervals. Although eHUBS replacement of walking and cycling slightly increases emissions in short-distance travel, this increase represents a negligible proportion relative to the total carbon reduction and is therefore manageable without impacting overall emission benefits. Overall, by replacing 30%-35% of conventional travel modes, eHUBS achieves a 15%-18% reduction in total emissions within medium-short, medium-long and long-distance intervals, especially through the substitution of motor vehicle use, underscoring its critical role in optimising urban transport and advancing environmental sustainability.

These findings above provide several key implications for urban transport policy and sustainable mobility planning. First, the results suggest that local authorities should prioritise distance-specific deployment of eHUBS modes to maximise carbon savings. For instance, in short-distance areas [0–5 km), dense deployment of eHUBS stations equipped with e-bikes and e-cargo bikes near city centres and smaller bus terminals would facilitate seamless transfers and encourage modal shift from private cars. In the medium-short interval [5–10 km), eHUBS stations should be strategically located between major public transport hubs and residential or commercial zones, with appropriate provision of e-bikes and e-scooters to maximise synergy with existing networks. For long-distance trips [20 km+), deploying eHUBS at intercity transport nodes such as rail stations and airports—with mode-appropriate options like e-cars—can offer last-mile or full-trip alternatives. Second, the observed carbon rebound effects—especially substitution of walking and cycling by e-cars—highlight the need for regulatory interventions such as dynamic pricing or differentiated subsidies to prevent zero-carbon displacement. Third, equity considerations must be central to eHUBS expansion, ensuring broad access across socio-economic groups and geographies. Finally, policymakers should support the integration of lifecycle emissions monitoring and user behaviour tracking into planning frameworks to enable iterative, data-driven optimisation of eHUBS services.

However, it is important to acknowledge that this study has certain limitations. In particular, the quantification of carbon reduction did not account for lifecycle emissions associated with the manufacturing and relocation of shared electric mobility vehicles. Incorporating such emissions presents methodological challenges due to the difficulty of allocating manufacturing-related emissions to individual trips, and the lack of relocation data from eHUBS operators, especially at this early pilot stage in Northwest Europe. Future research should aim to address these gaps by developing a more comprehensive model that includes lifecycle and operational emissions, supported by detailed data from eHUBS service providers.

Additionally, this study did not include demographic factors as control variables in the mode substitution analysis. Although such factors, such as age, gender, and household income, play an important role in shaping travel behaviour, a detailed behavioural analysis incorporating these variables was beyond the scope of the present study. Future work should explore these behavioural mechanisms in depth using disaggregated data, in order to better understand how demographic differences influence the adoption and substitution effects of shared electric mobility modes. This direction will help enhance the accuracy of carbon reduction assessments and contribute to a fuller understanding of the environmental impacts of shared electric mobility systems.

Furthermore, we acknowledge that modelling based on respondents’ stated willingness to use eHUBS—captured through Likert-scale responses—may be subject to intention–behaviour gaps, potentially leading to overestimation of substitution rates and associated carbon reductions. To assess this, we conducted a sensitivity analysis using a more conservative set of probability values (0%–95%) in place of the original linear mapping. The results indicate a reduction of approximately 2% in overall substitution rates, confirming a degree of upward bias while demonstrating the robustness of the model under more cautious behavioural assumptions. Moreover, we recognise the need for future research to incorporate spatial characteristics—such as the proximity between eHUBS and public transport stops—as well as dynamic mode-switching behaviours across trip distances, purposes, and levels of accessibility. This would require the integration of large-scale travel surveys, operational datasets, GIS tools, and spatial–behavioural modelling techniques to develop more empirically grounded substitution frameworks with stronger relevance for real-world policy and deployment.

Moreover, the analysis did not incorporate food production emissions associated with walking and cycling trips. While we acknowledge that such emissions are relevant in a broader lifecycle context, including them is not feasible within the current study’s framework, which assumes walking and cycling to be zero-emission modes in line with prevailing sustainability and public health paradigms. Future research could consider extending the model to include food-energy-related emissions where appropriate.

Finally, this study has not accounted for detailed daily modal choice behaviour and specific behavioural constraints, e.g., walking distances to eHUBS, service frequency, and accessibility factors. Although these behavioural realities are critical for accurately capturing substitution effects and emissions reductions, the necessary detailed behavioural data collection was limited during our survey, partly due to logistical constraints imposed by the COVID-19 pandemic period (2021–2023). Future research will prioritise the integration of these behavioural constraints and realism by leveraging detailed behavioural datasets such as the UK National Travel Survey, thereby refining the analytical robustness and practical applicability of findings.

## Supplementary Information

Below is the link to the electronic supplementary material.

Supplementary Material 1

## Data Availability

The datasets analysed in this study are not publicly available due to ethical and contractual restrictions with data providers (e.g., Transport for Greater Manchester). Access may be granted upon request to the corresponding author, subject to approval by the original data owners.
