# Supplementary material for: Quantifying carbon reductions from mode substitution through shared electric mobility hubs in Greater Manchester
Source: Sci Rep. 2025 Nov 5;15:38815. doi: 10.1038/s41598-025-22719-3 (PMC12589470; doi:10.1038/s41598-025-22719-3)
Supplement: Supplementary file 1 — Supplementary Material 1 [file 41598_2025_22719_MOESM1_ESM.docx]

**Appendix A**

**Shared Mobility User Experience Survey**

**I. Study background**

In this online survey, funded by the Interreg North-West Europe eHUBS project, we are interested in your use of shared mobility options, defined as the access to shared conventional or electric vehicles on an as-needed basis. Here, we are particularly interested in the use of shared vehicles available at electric mobility hubs, also known as eHUBs. eHUBS are on-street locations in local neighbourhoods, or at bus or train stations, that offer members of the public access to a range of shared electric vehicles including e-bikes, e-cargo bikes, and e-cars (see Figure 1 for an illustration). eHUBS are for everybody and can fulfil different travel needs by providing shared mobility options for a range of situations, be it for the daily commute, grocery shopping, or leisure.


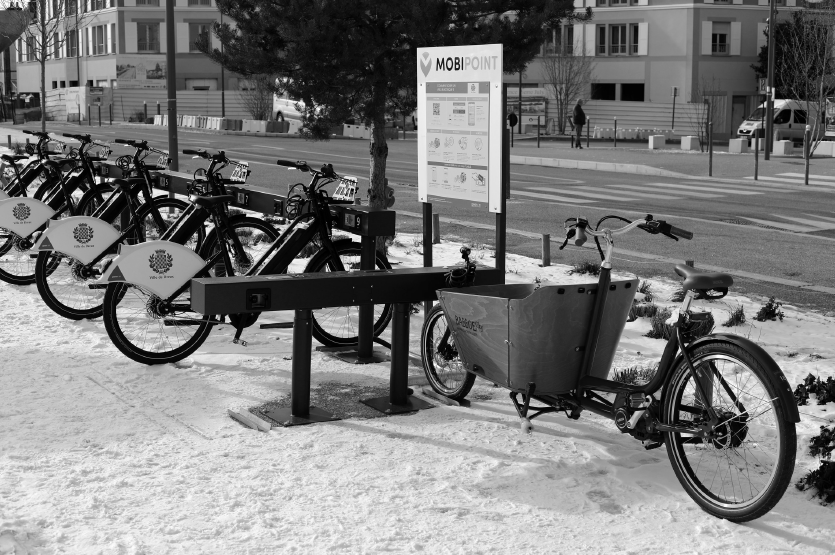

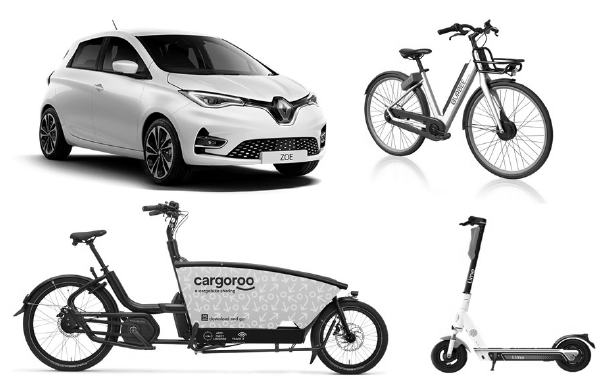


Figure 1. Illustration of an electric mobility hub (eHUB).

**II. Study participation and Consent**

Here, we would like to know about **your** experience with shared mobility. You will be asked to provide us with some basic information about yourself, some details about your general travel behaviour, as well as your shared mobility use and attitudes. All provided information will be kept strictly confidential and used for research purposes only.

**1. To continue with the survey, please enter your unique* ***Prolific ID*** *below.*

|  |
| --- |

As mentioned on the previously, we will ask you to provide some basic information about yourself, information about your general travel behaviour, and use of shared electric vehicles. If you do not wish to give an answer to a specific question, except required ones, you can leave it blank.

**2. If you wish to take part in this survey, please confirm that you are 18 years or over*

|  | *I hereby confirm that I am 18 years or over.* |
| --- | --- |

**3. Consent*

|  | *I hereby confirm that my participation in this survey is entirely voluntary, that I have been adequately informed about the purpose of the study, and that I can withdraw my participation from this survey at any time for any reason.* |
| --- | --- |
|  | *I am aware that, at any time, I have the right to request the survey administrators for copies of my personal data or to delete my personal data.* |
|  | *By consenting to this privacy notice, I give permission for my personal data to be processed for the purposes identified.* |

**III. About you**

**4. What is your age?*

| 18 to 24 | 25 to 34 | 35 to 44 | 45 to 54 | 55 to 64 | 65 to 74 | 75 or older |
| --- | --- | --- | --- | --- | --- | --- |
|  |  |  |  |  |  |  |

**5.*What is your gender?

| Female | MaleE | Other (please specify) |
| --- | --- | --- |
|  |  |  |

**6. What is your city of residence?*

|  |
| --- |

**7. Including yourself, how many* ***adults*** *usually live in your household?*

|  |
| --- |

**8. How many children usually live in your household?*

|  |
| --- |

**9. What is the highest level of education that you have completed?*

| No school education | Primary school education | Secondary school education |
| --- | --- | --- |
| Professional qualification | Undergraduate studies | Postgraduate studies |
| Prefer not to say |  |  |

**10. Please describe your current occupation.*

|  |
| --- |

**11. Approximately, what is your gross annual household income?*

| < £20,000 / 24.000€ | £20,000-£39,999 / 24.000-47.999€ |
| --- | --- |
| £40,000-£59,999 / 48.000-71.999€ | £60,000-£79,999 / 72.000-95.999€ |
| £80,000-£99,999 / 96.000-120.000€ | > £100,000 / 120.000€ |
| Prefer not to say |  |

**IV. Car details**

For the purpose of emission calculations, we would like to know a few details about the car that you use most often. Please provide the following information to the best of your knowledge.

**12. Are you usually the driver or passenger?*

| Driver | Passenger | Regularly switching between being the driver or passenger |
| --- | --- | --- |

**13. How many passengers are usually travelling with you?*

| 0 | 1 | 2 | 3 | 4 |
| --- | --- | --- | --- | --- |

**14. Please tell us the make and model of the car you use most often (e.g., ‘BMW M4’, ‘Fiat*

*500’ or ‘Range Rover’).*

|  |
| --- |

**15. What kind of fuel does this car run on?*

| Petrol | Diesel | Hybrid | Electric | Other (please specify) |
| --- | --- | --- | --- | --- |

**V. Regular trip details and alternatives**

On this page, we would like you to detail the steps of your commute to work, school/university or other regular commitment. You will then be presented with four different alternatives to complete this trip. For these alternatives, please select towhat extent you think they could be a suitable replacement for your current trip.

**16. Please outline the individual steps of your most regular trip (door to door, one-way only,*

*including waiting times).*

|  | Travel mode | Duration in minutes |
| --- | --- | --- |
| Step 1 |  |  |
| Step 2 |  |  |
| Step 3 |  |  |
| Step 4 |  |  |
| Step 5 |  |  |
| Step 6 |  |  |
| Step 7 |  |  |

**17. What is the approximate distance of this trip in miles? If you are unsure, please provide your closest estimate.*

|  |
| --- |

**18. What are the first 4 postcode digits of the area where your regular trip begins (origin)?*

|  |
| --- |

**19. What are the first 4 postcode digits of the area where your regular trip ends (destination)?*

|  |
| --- |

**20. How often do you make this trip?*

| On 5 days per week or more | On 3-4 days per week |
| --- | --- |
| On 1-2 days per week | 2-3 times per month |
| Once per month or less |  |

**VI. eHUBS mode substitution**

**21. If eHUBS were available in your city, to what extent would you be willing to use the*

*following alternatives for this trip?*

|  | I would not use it for any trips of this purpose | I may use it for a few trips of this purpose | I may use it for many trips of this purpose | I may use it for most trips of this purpose | I may use it for all trips of this purpose |
| --- | --- | --- | --- | --- | --- |
| **Electric car** for the entire trip |  |  |  |  |  |
| **Electric car** in combination with public transport |  |  |  |  |  |
| **Electric bike** for the entire trip |  |  |  |  |  |
| **Electric bike** in combination with public transport |  |  |  |  |  |
| **Electric cargo bike** for the entire trip |  |  |  |  |  |
| **Electric cargo bike** in combination with public transport |  |  |  |  |  |
| **Electric scooter** for the entire trip |  |  |  |  |  |
| **Electric scooter**  in combination with public transport |  |  |  |  |  |

Thank you very much for your participation! Please click the 'Done' button below to save your responses.

**Appendix B**

**Table I.** The various probabilities for e-car $(k=1)$ substituting conventional travel combinations across each distance interval group.

|  |  | $P\left( G_{i}H_{j} \right)$ | $\bar{P}\left( E_{1}\vert G_{i}H_{j} \right)$ | $\tilde{P}(E_{1}\vert G_{i}H_{j})$ | $P(E_{1}G_{i}H_{j})$ | $P(E_{1}G_{i}\vert H_{j})$ | $\bar{P}\left( F_{1}\vert E_{1}G_{i}H_{j} \right)$ | $P(F_{1}E_{1}G_{i}H_{j})$ | $P(F_{1}E_{1}G_{i}\vert H_{j})$ | $P\left[ (1-F_{1})E_{1}G_{i}\vert H_{j} \right]$ |
| --- | --- | --- | --- | --- | --- | --- | --- | --- | --- | --- |
| $H_{1}$ | $G_{1}$ | 13.70% | 35.10% | 8.77% | 1.20% | 3.69% | 38.14% | 0.46% | 1.18% | 1.92% |
|  | $G_{2}$ | 2.98% | 11.03% | 2.76% | 0.08% | 1.03% | 33.09% | 0.03% | 0.07% | 0.14% |
|  | $G_{3}$ | 22.04% | 11.25% | 2.81% | 0.62% | 6.35% | 12.95% | 0.08% | 0.21% | 1.39% |
| $H_{2}$ | $G_{1}$ | 20.63% | 38.19% | 9.55% | 1.97% | 6.47% | 29.68% | 0.58% | 2.26% | 5.33% |
|  | $G_{2}$ | 4.66% | 14.15% | 3.54% | 0.16% | 1.50% | 11.32% | 0.02% | 0.07% | 0.55% |
|  | $G_{3}$ | 0.70% | 3.13% | 0.78% | 0.01% | 0.27% | 6.25% | 0.00% | 0.00% | 0.04% |
| $H_{3}$ | $G_{1}$ | 14.75% | 39.58% | 9.90% | 1.46% | 5.70% | 22.92% | 0.33% | 1.69% | 5.67% |
|  | $G_{2}$ | 4.65% | 30.19% | 7.55% | 0.35% | 1.66% | 20.75% | 0.07% | 0.37% | 1.40% |
|  | $G_{3}$ | 0.44% | 20.00% | 5.00% | 0.02% | 0.15% | 5.00% | 0.00% | 0.01% | 0.10% |
| $H_{4}$ | $G_{1}$ | 12.90% | 40.99% | 10.25% | 1.32% | 3.11% | 14.80% | 0.20% | 1.26% | 7.28% |
|  | $G_{2}$ | 2.46% | 37.50% | 9.38% | 0.23% | 1.36% | 35.71% | 0.08% | 0.54% | 0.96% |
|  | $G_{3}$ | 0.09% | 0.00% | 0.00% | 0.00% | 0.00% | 0.00% | 0.00% | 0.00% | 0.00% |

**Table II**. The various probabilities for e-bike $(k=2)$ substituting conventional travel combinations across each distance interval group.

|  |  | $P\left( G_{i}H_{j} \right)$ | $\bar{P}\left( E_{2}\vert G_{i}H_{j} \right)$ | $\tilde{P}(E_{2}\vert G_{i}H_{j})$ | $P(E_{2}G_{i}H_{j})$ | $P(E_{2}G_{i}\vert H_{j})$ | $\bar{P}\left( F_{2}\vert E_{2}G_{i}H_{j} \right)$ | $P(F_{2}E_{2}G_{i}H_{j})$ | $P(F_{2}E_{2}G_{i}\vert H_{j})$ | $P\left[ (1-F_{2})E_{2}G_{i}\vert H_{j} \right]$ |
| --- | --- | --- | --- | --- | --- | --- | --- | --- | --- | --- |
| $H_{1}$ | $G_{1}$ | 13.70% | 41.83% | 10.46% | 1.43% | 3.69% | 66.03% | 0.94% | 2.44% | 1.25% |
|  | $G_{2}$ | 2.98% | 53.68% | 13.42% | 0.40% | 1.03% | 66.18% | 0.26% | 0.68% | 0.35% |
|  | $G_{3}$ | 22.04% | 44.72% | 11.18% | 2.46% | 6.35% | 56.47% | 1.39% | 3.59% | 2.77% |
| $H_{2}$ | $G_{1}$ | 20.63% | 32.55% | 8.14% | 1.68% | 6.47% | 62.98% | 1.06% | 4.07% | 2.39% |
|  | $G_{2}$ | 4.66% | 33.49% | 8.37% | 0.39% | 1.50% | 64.15% | 0.25% | 0.96% | 0.54% |
|  | $G_{3}$ | 0.70% | 37.50% | 9.38% | 0.07% | 0.27% | 53.13% | 0.04% | 0.14% | 0.13% |
| $H_{3}$ | $G_{1}$ | 14.75% | 30.65% | 7.66% | 1.13% | 5.70% | 61.01% | 0.69% | 3.48% | 2.22% |
|  | $G_{2}$ | 4.65% | 28.77% | 7.19% | 0.33% | 1.66% | 73.58% | 0.24% | 1.22% | 0.44% |
|  | $G_{3}$ | 0.44% | 30.00% | 7.50% | 0.03% | 0.15% | 45.00% | 0.01% | 0.07% | 0.07% |
| $H_{4}$ | $G_{1}$ | 12.90% | 14.80% | 3.70% | 0.48% | 3.11% | 67.18% | 0.32% | 2.09% | 1.02% |
|  | $G_{2}$ | 2.46% | 34.82% | 8.71% | 0.21% | 1.36% | 73.21% | 0.15% | 1.00% | 0.36% |
|  | $G_{3}$ | 0.09% | 0.00% | 0.00% | 0.00% | 0.00% | 25.00% | 0.00% | 0.00% | 0.00% |

**Table III**. The various probabilities for e-cargo bike $(k=3)$ substituting conventional travel combinations across each distance interval group.

|  |  | $P\left( G_{i}H_{j} \right)$ | $\bar{P}\left( E_{3}\vert G_{i}H_{j} \right)$ | $\tilde{P}(E_{3}\vert G_{i}H_{j})$ | $P(E_{3}G_{i}H_{j})$ | $P(E_{3}G_{i}\vert H_{j})$ | $\bar{P}\left( F_{3}\vert E_{3}G_{i}H_{j} \right)$ | $P(F_{3}E_{3}G_{i}H_{j})$ | $P(F_{3}E_{3}G_{i}\vert H_{j})$ | $P\left[ (1-F_{3})E_{3}G_{i}\vert H_{j} \right]$ |
| --- | --- | --- | --- | --- | --- | --- | --- | --- | --- | --- |
| $H_{1}$ | $G_{1}$ | 13.70% | 24.52% | 6.13% | 0.84% | 2.17% | 33.01% | 0.28% | 0.72% | 1.45% |
|  | $G_{2}$ | 2.98% | 41.18% | 10.29% | 0.31% | 0.80% | 66.18% | 0.21% | 0.53% | 0.27% |
|  | $G_{3}$ | 22.04% | 31.47% | 7.87% | 1.73% | 4.47% | 39.54% | 0.68% | 1.77% | 2.70% |
| $H_{2}$ | $G_{1}$ | 20.63% | 23.94% | 5.98% | 1.23% | 4.74% | 46.17% | 0.57% | 2.19% | 2.55% |
|  | $G_{2}$ | 4.66% | 46.23% | 11.56% | 0.54% | 2.08% | 69.34% | 0.37% | 1.44% | 0.64% |
|  | $G_{3}$ | 0.70% | 21.88% | 5.47% | 0.04% | 0.16% | 34.38% | 0.01% | 0.05% | 0.10% |
| $H_{3}$ | $G_{1}$ | 14.75% | 37.05% | 9.26% | 1.37% | 6.90% | 55.36% | 0.76% | 3.82% | 3.08% |
|  | $G_{2}$ | 4.65% | 43.87% | 10.97% | 0.51% | 2.57% | 70.28% | 0.36% | 1.81% | 0.76% |
|  | $G_{3}$ | 0.44% | 20.00% | 5.00% | 0.02% | 0.10% | 20.00% | 0.00% | 0.02% | 0.07% |
| $H_{4}$ | $G_{1}$ | 12.90% | 36.39% | 9.10% | 1.17% | 7.57% | 0.81% | 0.81% | 5.24% | 2.33% |
|  | $G_{2}$ | 2.46% | 43.75% | 10.94% | 0.27% | 1.75% | 0.21% | 0.21% | 1.37% | 0.37% |
|  | $G_{3}$ | 0.09% | 0.00% | 0.00% | 0.00% | 0.00% | 0.00% | 0.00% | 0.00% | 0.00% |

**Table IV**. The various probabilities for e-scooter $(k=4)$ substituting conventional travel combinations across each distance interval group.

|  |  | $P\left( G_{i}H_{j} \right)$ | $\bar{P}\left( E_{4}\vert G_{i}H_{j} \right)$ | $\tilde{P}(E_{4}\vert G_{i}H_{j})$ | $P(E_{4}G_{i}H_{j})$ | $P(E_{4}G_{i}\vert H_{j})$ | $\bar{P}\left( F_{4}\vert E_{4}G_{i}H_{j} \right)$ | $P(F_{4}E_{4}G_{i}H_{j})$ | $P(F_{4}E_{4}G_{i}\vert H_{j})$ | $P\left[ (1-F_{4})E_{4}G_{i}\vert H_{j} \right]$ |
| --- | --- | --- | --- | --- | --- | --- | --- | --- | --- | --- |
| $H_{1}$ | $G_{1}$ | 13.70% | 31.09% | 7.77% | 1.06% | 2.74% | 68.91% | 0.73% | 6.23% | 5.47% |
|  | $G_{2}$ | 2.98% | 33.09% | 8.27% | 0.25% | 0.65% | 71.32% | 0.18% | 1.74% | 0.95% |
|  | $G_{3}$ | 22.04% | 30.78% | 7.69% | 1.69% | 4.36% | 62.65% | 1.06% | 8.30% | 8.48% |
| $H_{2}$ | $G_{1}$ | 20.63% | 28.40% | 7.10% | 1.46% | 5.62% | 60.74% | 0.89% | 11.93% | 12.48% |
|  | $G_{2}$ | 4.66% | 30.19% | 7.55% | 0.35% | 1.35% | 63.68% | 0.22% | 3.33% | 2.22% |
|  | $G_{3}$ | 0.70% | 25.00% | 6.25% | 0.04% | 0.16% | 21.88% | 0.01% | 0.22% | 0.39% |
| $H_{3}$ | $G_{1}$ | 14.75% | 33.78% | 8.44% | 1.24% | 6.25% | 70.39% | 0.87% | 13.39% | 12.82% |
|  | $G_{2}$ | 4.65% | 33.49% | 8.37% | 0.39% | 1.97% | 61.79% | 0.24% | 4.61% | 3.35% |
|  | $G_{3}$ | 0.44% | 10.00% | 2.50% | 0.01% | 0.05% | 70.00% | 0.01% | 0.14% | 0.26% |
| $H_{4}$ | $G_{1}$ | 12.90% | 27.72% | 6.93% | 0.89% | 5.76% | 68.88% | 0.61% | 12.56% | 12.42% |
|  | $G_{2}$ | 2.46% | 50.89% | 12.72% | 0.31% | 2.00% | 58.93% | 0.18% | 4.09% | 2.51% |
|  | $G_{3}$ | 0.09% | 0.00% | 0.00% | 0.00% | 0.00% | 25.00% | 0.00% | 0.00% | 0.00% |

**Appendix C. Sensitivity Analysis of Likert-Scale Probability Mapping**

To assess the robustness of the baseline probability mapping (0%, 25%, 50%, 75%, 100%) used for converting Likert-scale responses into numeric values, a set of five alternative probability scenarios was introduced. These scenarios were designed to reflect a wide range of plausible behavioural interpretations, including minor boundary adjustments, more optimistic or pessimistic assumptions, and redistributions toward central tendencies.

The first scenario, Trimmed-extremes (5%, 25%, 50%, 75%, 95%), was designed to test whether replacing the extreme values of 0% and 100% with 5% and 95% would meaningfully affect the results, given that extreme responses are relatively rare in survey data. The second scenario, Optimistic (0%, 20%, 55%, 80%, 99%), slightly increased the mid- and high-level probabilities to reflect a more favourable behavioural assumption, while capping the maximum at 99% to avoid absolute certainty. The third scenario, Conservative (0%, 20%, 45%, 70%, 95%), uniformly shifted down non-zero categories by approximately five percentage points, representing a more cautious interpretation of self-reported intentions. The fourth scenario, Central tendency (10%, 30%, 50%, 70%, 90%), redistributed probability mass by raising the lower categories and lowering the higher ones, thereby generating an offsetting effect that emphasises moderation and reduces the impact of extreme or highly confident responses. Finally, the fifth scenario, Pessimistic (0%, 10%, 30%, 60%, 85%), applied substantial downward adjustments across all non-zero categories, particularly reducing mid- and high-level values, to stress-test the robustness of results under the most unfavourable behavioural assumptions.

The comparative results for overall eHUBS substitution rates under these alternative scenarios are summarised in Table V. Conditional formatting was applied to facilitate interpretation: green indicates positive deviations (alternative > baseline), red indicates negative deviations (alternative < baseline), and colour intensity reflects the magnitude of deviation. Across the five scenarios, the differences relative to the baseline mapping are small. The Trimmed-extremes scenario produced the smallest deviations, all within ±1%, suggesting negligible influence from boundary adjustments. The Optimistic scenario yielded slightly higher substitution rates, with most increases confined to +0.06% to +0.6%. The Conservative scenario produced moderate downward deviations, typically between –0.1% and –1.2%, with a maximum close to –2.5%. The Central tendency scenario exhibited offsetting effects, with most deviations ranging between –0.8% and +0.7%. The Pessimistic scenario generated the largest downward shifts, concentrated between –1.0% and –2.85%, with no deviation exceeding three percentage points. These findings confirm that even under extreme assumptions, the conclusions regarding overall substitution rates remain stable.

In addition to the aggregate results, the mode-specific substitution probabilities under each scenario are presented in Tables VI–X. These tables provide a more granular view by comparing the probabilities of individual eHUBS modes with the baseline mapping. The results further support the stability of the model. Specifically, the Trimmed-extremes scenario showed the smallest changes, typically within –0.9% to +0.6%, with very few cases approaching ±1.6%. The Optimistic scenario produced slight upward shifts, generally between –0.2% and +0.6%. The Conservative scenario displayed more noticeable downward changes, ranging from –0.1% and –1.2% in most cases, and occasionally approaching –2.5%. The Central tendency scenario showed limited variation, with most differences within –0.8% and +0.7%. Finally, the Pessimistic scenario resulted in the largest reductions, with most deviations between –0.5% and –1.7%, and only isolated cases exceeding –2%.

Taken together, the results from Tables V–X demonstrate that the overall eHUBS substitution rates and mode-specific estimates are stable across a wide range of alternative probability assignments. Although the five scenarios introduce slight directional differences—optimistic scenarios yielding upward deviations, conservative and pessimistic scenarios producing downward deviations, central tendency scenarios remaining neutral, and trimmed-extremes scenarios showing negligible changes—the magnitudes of these shifts remain modest. This confirms that the baseline mapping (0%, 25%, 50%, 75%, 100%) is not unduly sensitive to arbitrary threshold choices, thereby reinforcing the robustness of the substitution analysis.

**Table V**. Differences in overall eHUBS substitution rates under five alternative Likert-scale probability mapping scenarios compared with the baseline mapping (0%, 25%, 50%, 75%, 100%), with conditional formatting indicating direction and magnitude of deviation.

| **Five Alternative Likert-scale Probability Mapping Scenarios** | **Hj** | **Gi** | **Alternative Likert-scale Mapping (for Sensitivity Analysis)** | **Baseline Likert-scale Mapping (0%, 25%, 50%, 75%, 100%)** | **Difference in eHUBS Substitution Rate (Alternative – Baseline)** |
| --- | --- | --- | --- | --- | --- |
| **Scenario 1**: Trimmed-extremes (i.e., 5%、25%、50%、75%、95%) | H1 | G1 | 10.77% | 11.70% | -0.93% |
|  |  | G2 | 2.46% | 2.69% | -0.23% |
|  |  | G3 | 16.52% | 16.78% | -0.26% |
|  | H2 | G1 | 24.97% | 24.40% | 0.57% |
|  |  | G2 | 4.92% | 5.54% | -0.62% |
|  |  | G3 | 0.72% | 0.63% | 0.09% |
|  | H3 | G1 | 25.32% | 26.21% | -0.89% |
|  |  | G2 | 7.48% | 7.96% | -0.48% |
|  |  | G3 | 0.38% | 0.40% | -0.02% |
|  | H4 | G1 | 25.84% | 24.98% | 0.86% |
|  |  | G2 | 5.69% | 6.60% | -0.91% |
|  |  | G3 | 0.00% | 0.00% | 0.00% |
| **Scenario 2**: Optimistic  (i.e., 0%、20%、55%、80%、99%) | H1 | G1 | 12.30% | 11.70% | 0.60% |
|  |  | G2 | 2.78% | 2.69% | 0.09% |
|  |  | G3 | 18.01% | 16.78% | 1.23% |
|  | H2 | G1 | 25.65% | 24.40% | 1.25% |
|  |  | G2 | 5.71% | 5.54% | 0.17% |
|  |  | G3 | 0.66% | 0.63% | 0.03% |
|  | H3 | G1 | 26.38% | 26.21% | 0.17% |
|  |  | G2 | 8.32% | 7.96% | 0.36% |
|  |  | G3 | 0.51% | 0.40% | 0.11% |
|  | H4 | G1 | 25.20% | 24.98% | 0.22% |
|  |  | G2 | 6.66% | 6.60% | 0.06% |
|  |  | G3 | 0.00% | 0.00% | 0.00% |
| **Scenario 3**: Conservative  (i.e., 0%、20%、45%、70%、95%) | H1 | G1 | 9.91% | 11.70% | -1.79% |
|  |  | G2 | 1.36% | 2.69% | -1.33% |
|  |  | G3 | 15.40% | 16.78% | -1.38% |
|  | H2 | G1 | 22.79% | 24.40% | -1.61% |
|  |  | G2 | 4.73% | 5.54% | -0.81% |
|  |  | G3 | 0.52% | 0.63% | -0.11% |
|  | H3 | G1 | 25.03% | 26.21% | -1.18% |
|  |  | G2 | 6.72% | 7.96% | -1.24% |
|  |  | G3 | 0.27% | 0.40% | -0.13% |
|  | H4 | G1 | 23.25% | 24.98% | -1.73% |
|  |  | G2 | 5.11% | 6.60% | -1.49% |
|  |  | G3 | 0.00% | 0.00% | 0.00% |
| **Scenario 4**:  Central tendency  (i.e., 10%、30%、50%、70%、90%) | H1 | G1 | 11.33% | 11.70% | -0.37% |
|  |  | G2 | 2.61% | 2.69% | -0.08% |
|  |  | G3 | 16.08% | 16.78% | -0.70% |
|  | H2 | G1 | 45.15% | 24.40% | -0.25% |
|  |  | G2 | 4.94% | 5.54% | -0.60% |
|  |  | G3 | 0.43% | 0.63% | -0.20% |
|  | H3 | G1 | 25.73% | 26.21% | -0.48% |
|  |  | G2 | 6.97% | 7.96% | -0.99% |
|  |  | G3 | 0.35% | 0.40% | -0.05% |
|  | H4 | G1 | 24.05% | 24.98% | -0.93% |
|  |  | G2 | 5.87% | 6.60% | -0.73% |
|  |  | G3 | 0.00% | 0.00% | 0.00% |
| **Scenario 5**: Pessimistic  (i.e., 0%、10%、30%、60%、85%) | H1 | G1 | 8.85% | 11.70% | -2.85% |
|  |  | G2 | 1.68% | 2.69% | -1.01% |
|  |  | G3 | 14.03% | 16.78% | -2.75% |
|  | H2 | G1 | 22.52% | 24.40% | -1.88% |
|  |  | G2 | 3.39% | 5.54% | -2.15% |
|  |  | G3 | 0.29% | 0.63% | -0.34% |
|  | H3 | G1 | 24.56% | 26.21% | -1.65% |
|  |  | G2 | 6.20% | 7.96% | -1.76% |
|  |  | G3 | 0.15% | 0.40% | -0.25% |
|  | H4 | G1 | 23.05% | 24.98% | -1.93% |
|  |  | G2 | 5.35% | 6.60% | -1.25% |
|  |  | G3 | 0.00% | 0.00% | 0.00% |

**Table VI.** Substitution probabilities of individual eHUBS modes under **Scenario 1** (Trimmed-extremes: 5%, 25%, 50%, 75%, 95%), compared with baseline mapping (0%, 25%, 50%, 75%, 100%).

| ***P(EkGi\|Hj)*** | *Alternative* | *Baseline* | *Difference* | ***P(EkGi\|Hj)*** | *Alternative* | *Baseline* | *Difference* |
| --- | --- | --- | --- | --- | --- | --- | --- |
| P(E1G1\|H1) | 2.84% | 3.10% | -0.26% | P(E1G1\|H3) | 6.51% | 7.36% | -0.85% |
| P(E2G1\|H1) | 3.43% | 3.69% | -0.26% | P(E2G1\|H3) | 5.15% | 5.70% | -0.55% |
| P(E3G1\|H1) | 1.99% | 2.17% | -0.18% | P(E3G1\|H3) | 6.10% | 6.90% | -0.80% |
| P(E4G1\|H1) | 2.51% | 2.74% | -0.23% | P(E4G1\|H3) | 7.56% | 6.25% | 1.31% |
| P(E1G2\|H1) | 0.18% | 0.21% | -0.03% | P(E1G2\|H3) | 1.34% | 1.76% | -0.42% |
| P(E2G2\|H1) | 0.96% | 1.03% | -0.07% | P(E2G2\|H3) | 2.17% | 1.66% | 0.51% |
| P(E3G2\|H1) | 0.74% | 0.80% | -0.06% | P(E3G2\|H3) | 2.41% | 2.57% | -0.16% |
| P(E4G2\|H1) | 0.58% | 0.65% | -0.07% | P(E4G2\|H3) | 1.56% | 1.97% | -0.41% |
| P(E1G3\|H1) | 1.66% | 1.60% | 0.06% | P(E1G3\|H3) | 0.08% | 0.10% | -0.02% |
| P(E2G3\|H1) | 5.90% | 6.35% | -0.45% | P(E2G3\|H3) | 0.16% | 0.15% | 0.01% |
| P(E3G3\|H1) | 4.66% | 4.47% | 0.19% | P(E3G3\|H3) | 0.10% | 0.10% | 0.00% |
| P(E4G3\|H1) | 4.31% | 4.36% | -0.05% | P(E4G3\|H3) | 0.05% | 0.05% | 0.00% |
| P(E1G1\|H2) | 6.88% | 7.58% | -0.70% | P(E1G1\|H4) | 8.06% | 8.54% | -0.48% |
| P(E2G1\|H2) | 5.87% | 6.47% | -0.60% | P(E2G1\|H4) | 6.63% | 3.11% | 3.52% |
| P(E3G1\|H2) | 6.26% | 4.74% | 1.52% | P(E3G1\|H4) | 6.28% | 7.57% | -1.29% |
| P(E4G1\|H2) | 5.96% | 5.62% | 0.34% | P(E4G1\|H4) | 4.88% | 5.76% | -0.88% |
| P(E1G2\|H2) | 0.48% | 0.62% | -0.14% | P(E1G2\|H4) | 1.02% | 1.49% | -0.47% |
| P(E2G2\|H2) | 1.23% | 1.50% | -0.27% | P(E2G2\|H4) | 1.34% | 1.36% | -0.02% |
| P(E3G2\|H2) | 1.98% | 2.08% | -0.10% | P(E3G2\|H4) | 1.84% | 1.75% | 0.09% |
| P(E4G2\|H2) | 1.22% | 1.35% | -0.13% | P(E4G2\|H4) | 1.48% | 2.00% | -0.52% |
| P(E1G3\|H2) | 0.22% | 0.08% | 0.14% | P(E1G3\|H4) | 0.00% | 0.00% | 0.00% |
| P(E2G3\|H2) | 0.22% | 0.27% | -0.05% | P(E2G3\|H4) | 0.00% | 0.00% | 0.00% |
| P(E3G3\|H2) | 0.13% | 0.16% | -0.03% | P(E3G3\|H4) | 0.00% | 0.00% | 0.00% |
| P(E4G3\|H2) | 0.15% | 0.16% | -0.01% | P(E4G3\|H4) | 0.00% | 0.00% | 0.00% |

**Table VII**. Substitution probabilities of individual eHUBS modes under **Scenario 2** (Optimistic: 0%, 20%, 55%, 80%, 99%), compared with baseline mapping (0%, 25%, 50%, 75%, 100%).

| ***P(EkGi\|Hj)*** | *Alternative* | *Baseline* | *Difference* | ***P(EkGi\|Hj)*** | *Alternative* | *Baseline* | *Difference* |
| --- | --- | --- | --- | --- | --- | --- | --- |
| P(E1G1\|H1) | 3.21% | 3.10% | 0.11% | P(E1G1\|H3) | 7.23% | 7.36% | -0.13% |
| P(E2G1\|H1) | 3.80% | 3.69% | 0.11% | P(E2G1\|H3) | 5.90% | 5.70% | 0.20% |
| P(E3G1\|H1) | 2.39% | 2.17% | 0.22% | P(E3G1\|H3) | 6.88% | 6.90% | -0.02% |
| P(E4G1\|H1) | 2.90% | 2.74% | 0.16% | P(E4G1\|H3) | 6.37% | 6.25% | 0.12% |
| P(E1G2\|H1) | 0.28% | 0.21% | 0.07% | P(E1G2\|H3) | 1.61% | 1.76% | -0.15% |
| P(E2G2\|H1) | 1.03% | 1.03% | 0.00% | P(E2G2\|H3) | 1.44% | 1.66% | -0.22% |
| P(E3G2\|H1) | 0.81% | 0.80% | 0.01% | P(E3G2\|H3) | 2.64% | 2.57% | 0.07% |
| P(E4G2\|H1) | 0.67% | 0.65% | 0.02% | P(E4G2\|H3) | 1.83% | 1.97% | -0.14% |
| P(E1G3\|H1) | 2.14% | 1.60% | 0.54% | P(E1G3\|H3) | 0.13% | 0.10% | 0.03% |
| P(E2G3\|H1) | 6.47% | 6.35% | 0.12% | P(E2G3\|H3) | 0.18% | 0.15% | 0.03% |
| P(E3G3\|H1) | 4.74% | 4.47% | 0.27% | P(E3G3\|H3) | 0.13% | 0.10% | 0.03% |
| P(E4G3\|H1) | 4.66% | 4.36% | 0.30% | P(E4G3\|H3) | 0.08% | 0.05% | 0.03% |
| P(E1G1\|H2) | 7.71% | 7.58% | 0.13% | P(E1G1\|H4) | 7.83% | 8.54% | -0.71% |
| P(E2G1\|H2) | 6.77% | 6.47% | 0.30% | P(E2G1\|H4) | 4.54% | 3.11% | 1.43% |
| P(E3G1\|H2) | 5.18% | 4.74% | 0.44% | P(E3G1\|H4) | 7.09% | 7.57% | -0.48% |
| P(E4G1\|H2) | 5.99% | 5.62% | 0.37% | P(E4G1\|H4) | 5.73% | 5.76% | -0.03% |
| P(E1G2\|H2) | 0.70% | 0.62% | 0.08% | P(E1G2\|H4) | 1.25% | 1.49% | -0.24% |
| P(E2G2\|H2) | 1.43% | 1.50% | -0.07% | P(E2G2\|H4) | 1.54% | 1.36% | 0.18% |
| P(E3G2\|H2) | 2.14% | 2.08% | 0.06% | P(E3G2\|H4) | 2.10% | 1.75% | 0.35% |
| P(E4G2\|H2) | 1.43% | 1.35% | 0.08% | P(E4G2\|H4) | 1.76% | 2.00% | -0.24% |
| P(E1G3\|H2) | 0.05% | 0.08% | -0.03% | P(E1G3\|H4) | 0.00% | 0.00% | 0.00% |
| P(E2G3\|H2) | 0.26% | 0.27% | -0.01% | P(E2G3\|H4) | 0.00% | 0.00% | 0.00% |
| P(E3G3\|H2) | 0.17% | 0.16% | 0.01% | P(E3G3\|H4) | 0.00% | 0.00% | 0.00% |
| P(E4G3\|H2) | 0.18% | 0.16% | 0.02% | P(E4G3\|H4) | 0.00% | 0.00% | 0.00% |

**Table VIII.** Substitution probabilities of individual eHUBS modes under **Scenario 3** (Conservative: 10%、30%、50%、70%、90%), compared with baseline mapping (0%, 25%, 50%, 75%, 100%).

| ***P(EkGi\|Hj)*** | *Alternative* | *Baseline* | *Difference* | ***P(EkGi\|Hj)*** | *Alternative* | *Baseline* | *Difference* |
| --- | --- | --- | --- | --- | --- | --- | --- |
| P(E1G1\|H1) | 2.37% | 3.10% | -0.73% | P(E1G1\|H3) | 7.02% | 7.36% | -0.34% |
| P(E2G1\|H1) | 2.84% | 3.69% | -0.85% | P(E2G1\|H3) | 5.24% | 5.70% | -0.46% |
| P(E3G1\|H1) | 2.20% | 2.17% | 0.03% | P(E3G1\|H3) | 6.10% | 6.90% | -0.80% |
| P(E4G1\|H1) | 2.50% | 2.74% | -0.24% | P(E4G1\|H3) | 6.67% | 6.25% | 0.42% |
| P(E1G2\|H1) | 0.36% | 0.21% | 0.15% | P(E1G2\|H3) | 1.66% | 1.76% | -0.10% |
| P(E2G2\|H1) | 0.30% | 1.03% | -0.73% | P(E2G2\|H3) | 1.20% | 1.66% | -0.46% |
| P(E3G2\|H1) | 0.50% | 0.80% | -0.30% | P(E3G2\|H3) | 2.66% | 2.57% | 0.09% |
| P(E4G2\|H1) | 0.20% | 0.65% | -0.45% | P(E4G2\|H3) | 1.20% | 1.97% | -0.77% |
| P(E1G3\|H1) | 2.80% | 1.60% | 1.20% | P(E1G3\|H3) | 0.03% | 0.10% | -0.07% |
| P(E2G3\|H1) | 5.20% | 6.35% | -1.15% | P(E2G3\|H3) | 0.13% | 0.15% | -0.02% |
| P(E3G3\|H1) | 3.90% | 4.47% | -0.57% | P(E3G3\|H3) | 0.06% | 0.10% | -0.04% |
| P(E4G3\|H1) | 3.50% | 4.36% | -0.86% | P(E4G3\|H3) | 0.05% | 0.05% | 0.00% |
| P(E1G1\|H2) | 5.20% | 7.58% | -2.38% | P(E1G1\|H4) | 6.88% | 8.54% | -1.66% |
| P(E2G1\|H2) | 7.09% | 6.47% | 0.62% | P(E2G1\|H4) | 4.21% | 3.11% | 1.10% |
| P(E3G1\|H2) | 4.70% | 4.74% | -0.04% | P(E3G1\|H4) | 6.36% | 7.57% | -1.21% |
| P(E4G1\|H2) | 5.80% | 5.62% | 0.18% | P(E4G1\|H4) | 5.80% | 5.76% | 0.04% |
| P(E1G2\|H2) | 0.89% | 0.62% | 0.27% | P(E1G2\|H4) | 1.11% | 1.49% | -0.38% |
| P(E2G2\|H2) | 1.04% | 1.50% | -0.46% | P(E2G2\|H4) | 0.74% | 1.36% | -0.62% |
| P(E3G2\|H2) | 1.90% | 2.08% | -0.18% | P(E3G2\|H4) | 1.50% | 1.75% | -0.25% |
| P(E4G2\|H2) | 0.90% | 1.35% | -0.45% | P(E4G2\|H4) | 1.76% | 2.00% | -0.24% |
| P(E1G3\|H2) | 0.08% | 0.08% | 0.00% | P(E1G3\|H4) | 0.00% | 0.00% | 0.00% |
| P(E2G3\|H2) | 0.14% | 0.27% | -0.13% | P(E2G3\|H4) | 0.00% | 0.00% | 0.00% |
| P(E3G3\|H2) | 0.09% | 0.16% | -0.07% | P(E3G3\|H4) | 0.00% | 0.00% | 0.00% |
| P(E4G3\|H2) | 0.20% | 0.16% | 0.04% | P(E4G3\|H4) | 0.00% | 0.00% | 0.00% |

**Table IX.** Substitution probabilities of individual eHUBS modes under **Scenario 4** (Central tendency: 5%, 25%, 50%, 75%, 95%), compared with baseline mapping (0%, 25%, 50%, 75%, 100%).

| ***P(EkGi\|Hj)*** | *Alternative* | *Baseline* | *Difference* | ***P(EkGi\|Hj)*** | *Alternative* | *Baseline* | *Difference* |
| --- | --- | --- | --- | --- | --- | --- | --- |
| P(E1G1\|H1) | 3.27% | 3.10% | 0.17% | P(E1G1\|H3) | 6.18% | 7.36% | -1.18% |
| P(E2G1\|H1) | 2.84% | 3.69% | -0.85% | P(E2G1\|H3) | 6.25% | 5.70% | 0.55% |
| P(E3G1\|H1) | 2.59% | 2.17% | 0.42% | P(E3G1\|H3) | 7.93% | 6.90% | 1.03% |
| P(E4G1\|H1) | 2.63% | 2.74% | -0.11% | P(E4G1\|H3) | 5.37% | 6.25% | -0.88% |
| P(E1G2\|H1) | 0.76% | 0.21% | 0.55% | P(E1G2\|H3) | 1.78% | 1.76% | 0.02% |
| P(E2G2\|H1) | 0.81% | 1.03% | -0.22% | P(E2G2\|H3) | 1.97% | 1.66% | 0.31% |
| P(E3G2\|H1) | 0.60% | 0.80% | -0.20% | P(E3G2\|H3) | 2.01% | 2.57% | -0.56% |
| P(E4G2\|H1) | 0.44% | 0.65% | -0.21% | P(E4G2\|H3) | 1.22% | 1.97% | -0.75% |
| P(E1G3\|H1) | 2.23% | 1.60% | 0.63% | P(E1G3\|H3) | 0.09% | 0.10% | -0.01% |
| P(E2G3\|H1) | 4.88% | 6.35% | -1.47% | P(E2G3\|H3) | 0.13% | 0.15% | -0.02% |
| P(E3G3\|H1) | 4.47% | 4.47% | 0.00% | P(E3G3\|H3) | 0.09% | 0.10% | -0.01% |
| P(E4G3\|H1) | 4.50% | 4.36% | 0.14% | P(E4G3\|H3) | 0.03% | 0.05% | -0.02% |
| P(E1G1\|H2) | 4.57% | 7.58% | -3.01% | P(E1G1\|H4) | 5.93% | 8.54% | -2.61% |
| P(E2G1\|H2) | 6.30% | 6.47% | -0.17% | P(E2G1\|H4) | 4.97% | 3.11% | 1.86% |
| P(E3G1\|H2) | 6.38% | 4.74% | 1.64% | P(E3G1\|H4) | 5.90% | 7.57% | -1.67% |
| P(E4G1\|H2) | 6.90% | 5.62% | 1.28% | P(E4G1\|H4) | 7.25% | 5.76% | 1.49% |
| P(E1G2\|H2) | 1.36% | 0.62% | 0.74% | P(E1G2\|H4) | 1.77% | 1.49% | 0.28% |
| P(E2G2\|H2) | 0.97% | 1.50% | -0.53% | P(E2G2\|H4) | 1.17% | 1.36% | -0.19% |
| P(E3G2\|H2) | 1.67% | 2.08% | -0.41% | P(E3G2\|H4) | 1.59% | 1.75% | -0.16% |
| P(E4G2\|H2) | 0.94% | 1.35% | -0.41% | P(E4G2\|H4) | 1.34% | 2.00% | -0.66% |
| P(E1G3\|H2) | 0.07% | 0.08% | -0.01% | P(E1G3\|H4) | 0.00% | 0.00% | 0.00% |
| P(E2G3\|H2) | 0.16% | 0.27% | -0.11% | P(E2G3\|H4) | 0.00% | 0.00% | 0.00% |
| P(E3G3\|H2) | 0.10% | 0.16% | -0.06% | P(E3G3\|H4) | 0.00% | 0.00% | 0.00% |
| P(E4G3\|H2) | 0.10% | 0.16% | -0.06% | P(E4G3\|H4) | 0.00% | 0.00% | 0.00% |

**Table X.** Substitution probabilities of individual eHUBS modes under **Scenario 5** (Pessimistic: 0%、10%、30%、60%、85%), compared with baseline mapping (0%, 25%, 50%, 75%, 100%).

| ***P(EkGi\|Hj)*** | *Alternative* | *Baseline* | *Difference* | ***P(EkGi\|Hj)*** | *Alternative* | *Baseline* | *Difference* |
| --- | --- | --- | --- | --- | --- | --- | --- |
| P(E1G1\|H1) | 1.79% | 3.10% | -1.31% | P(E1G1\|H3) | 6.06% | 7.36% | -1.30% |
| P(E2G1\|H1) | 2.78% | 3.69% | -0.91% | P(E2G1\|H3) | 4.60% | 5.70% | -1.10% |
| P(E3G1\|H1) | 1.49% | 2.17% | -0.68% | P(E3G1\|H3) | 6.74% | 6.90% | -0.16% |
| P(E4G1\|H1) | 2.79% | 2.74% | 0.05% | P(E4G1\|H3) | 7.16% | 6.25% | 0.91% |
| P(E1G2\|H1) | 0.19% | 0.21% | -0.02% | P(E1G2\|H3) | 1.47% | 1.76% | -0.29% |
| P(E2G2\|H1) | 0.57% | 1.03% | -0.46% | P(E2G2\|H3) | 1.29% | 1.66% | -0.37% |
| P(E3G2\|H1) | 0.85% | 0.80% | 0.05% | P(E3G2\|H3) | 1.67% | 2.57% | -0.90% |
| P(E4G2\|H1) | 0.07% | 0.65% | -0.58% | P(E4G2\|H3) | 1.77% | 1.97% | -0.20% |
| P(E1G3\|H1) | 2.60% | 1.60% | 1.00% | P(E1G3\|H3) | 0.01% | 0.10% | -0.09% |
| P(E2G3\|H1) | 4.48% | 6.35% | -1.87% | P(E2G3\|H3) | 0.07% | 0.15% | -0.08% |
| P(E3G3\|H1) | 3.49% | 4.47% | -0.98% | P(E3G3\|H3) | 0.01% | 0.10% | -0.09% |
| P(E4G3\|H1) | 3.46% | 4.36% | -0.90% | P(E4G3\|H3) | 0.06% | 0.05% | 0.01% |
| P(E1G1\|H2) | 5.55% | 7.58% | -2.03% | P(E1G1\|H4) | 5.75% | 8.54% | -2.79% |
| P(E2G1\|H2) | 5.54% | 6.47% | -0.93% | P(E2G1\|H4) | 4.93% | 3.11% | 1.82% |
| P(E3G1\|H2) | 5.75% | 4.74% | 1.01% | P(E3G1\|H4) | 6.94% | 7.57% | -0.63% |
| P(E4G1\|H2) | 5.70% | 5.62% | 0.08% | P(E4G1\|H4) | 5.42% | 5.76% | -0.34% |
| P(E1G2\|H2) | 0.51% | 0.62% | -0.11% | P(E1G2\|H4) | 1.10% | 1.49% | -0.39% |
| P(E2G2\|H2) | 1.36% | 1.50% | -0.14% | P(E2G2\|H4) | 1.34% | 1.36% | -0.02% |
| P(E3G2\|H2) | 1.15% | 2.08% | -0.93% | P(E3G2\|H4) | 1.12% | 1.75% | -0.63% |
| P(E4G2\|H2) | 0.37% | 1.35% | -0.98% | P(E4G2\|H4) | 1.79% | 2.00% | -0.21% |
| P(E1G3\|H2) | 0.02% | 0.08% | -0.06% | P(E1G3\|H4) | 0.00% | 0.00% | 0.00% |
| P(E2G3\|H2) | 0.05% | 0.27% | -0.22% | P(E2G3\|H4) | 0.00% | 0.00% | 0.00% |
| P(E3G3\|H2) | 0.06% | 0.16% | -0.10% | P(E3G3\|H4) | 0.00% | 0.00% | 0.00% |
| P(E4G3\|H2) | 0.16% | 0.16% | 0.00% | P(E4G3\|H4) | 0.00% | 0.00% | 0.00% |

**Appendix D**

**Table XI**. The results of $T_{k11}^{wh}$, $T_{k11}^{pa}$, $M_{k11}^{pa}$ and $M_{k11}^{wh}$.

| $H_{j}$ | $G_{i}$ | $T_{kij}^{wh}, M_{kij}^{wh}, T_{kij}^{pa}, M_{kij}^{pa}$ | $k=1$ | $k=2$ | $k=3$ | $k=4$ | Total |
| --- | --- | --- | --- | --- | --- | --- | --- |
| $H_{1}$ | $G_{1}$ | $T_{k11}^{wh}$ | 3 | 2 | 2 | 1 | 8 |
|  |  | $M_{k11}^{wh}$ | 0.41 | 0.02 | 0.02 | 0.02 | 0.47 |
|  |  | $T_{k11}^{pa}$ | 2 | 4 | 1 | 3 | 10 |
|  |  | $M_{k11}^{pa}$ | 0.39 | 0.75 | 0.17 | 0.52 | 1.83 |
|  | $G_{2}$ | $T_{k21}^{wh}$ | 0 | 0 | 0 | 0 | 0 |
|  |  | $M_{k21}^{wh}$ | 0.00 | 0.00 | 0.00 | 0.00 | 0.00 |
|  |  | $T_{k21}^{pa}$ | 0 | 0 | 0 | 0 | 0 |
|  |  | $M_{k21}^{pa}$ | 0.00 | 0.00 | 0.00 | 0.00 | 0.00 |
|  | $G_{3}$ | $T_{k31}^{wh}$ | 3 | 7 | 7 | 4 | 21 |
|  |  | $M_{k31}^{wh}$ | 0.18 | 0.03 | 0.03 | 0.04 | 0.28 |
|  |  | $T_{k31}^{pa}$ | 1 | 9 | 4 | 7 | 21 |
|  |  | $M_{k31}^{pa}$ | 0.09 | 0.69 | 0.31 | 0.54 | 1.63 |
| $H_{2}$ | $G_{1}$ | $T_{k12}^{wh}$ | 13 | 6 | 6 | 5 | 30 |
|  |  | $M_{k12}^{wh}$ | 4.10 | 0.14 | 0.12 | 0.27 | 4.87 |
|  |  | $T_{k12}^{pa}$ | 5 | 10 | 5 | 8 | 28 |
|  |  | $M_{k12}^{pa}$ | 2.24 | 3.93 | 1.96 | 3.19 | 11.32 |
|  | $G_{2}$ | $T_{k22}^{wh}$ | 0 | 0 | 0 | 0 | 0 |
|  |  | $M_{k22}^{wh}$ | 0.00 | 0.00 | 0.00 | 0.00 | 0.00 |
|  |  | $T_{k22}^{pa}$ | 0 | 1 | 1 | 0 | 2 |
|  |  | $M_{k22}^{pa}$ | 0.00 | 0.43 | 0.43 | 0.00 | 0.86 |
|  | $G_{3}$ | $T_{k32}^{wh}$ | 0 | 0 | 0 | 0 | 0 |
|  |  | $M_{k32}^{wh}$ | 0.00 | 0.00 | 0.00 | 0.00 | 0.00 |
|  |  | $T_{k32}^{pa}$ | 0 | 0 | 0 | 0 | 0 |
|  |  | $M_{k32}^{pa}$ | 0.00 | 0.00 | 0.00 | 0.00 | 0.00 |
| $H_{3}$ | $G_{1}$ | $T_{k13}^{wh}$ | 10 | 4 | 5 | 3 | 22 |
|  |  | $M_{k13}^{wh}$ | 6.50 | 0.19 | 0.20 | 0.33 | 7.22 |
|  |  | $T_{k13}^{pa}$ | 3 | 6 | 6 | 7 | 22 |
|  |  | $M_{k13}^{pa}$ | 2.81 | 5.04 | 5.03 | 5.95 | 18.83 |
|  | $G_{2}$ | $T_{k23}^{wh}$ | 1 | 0 | 0 | 0 | 1 |
|  |  | $M_{k23}^{wh}$ | 0.61 | 0.00 | 0.00 | 0.00 | 0.61 |
|  |  | $T_{k23}^{pa}$ | 0 | 1 | 1 | 1 | 3 |
|  |  | $M_{k23}^{pa}$ | 0.00 | 0.79 | 0.79 | 0.80 | 2.38 |
|  | $G_{3}$ | $T_{k33}^{wh}$ | 0 | 0 | 0 | 0 | 0 |
|  |  | $M_{k33}^{wh}$ | 0.00 | 0.00 | 0.00 | 0.00 | 0.00 |
|  |  | $T_{k33}^{pa}$ | 0 | 0 | 0 | 0 | 0 |
|  |  | $M_{k33}^{pa}$ | 0.00 | 0.00 | 0.00 | 0.00 | 0.00 |
| $H_{4}$ | $G_{1}$ | $T_{k14}^{wh}$ | 11 | 1 | 3 | 3 | 18 |
|  |  | $M_{k14}^{wh}$ | 21.88 | 0.14 | 0.37 | 1.02 | 23.41 |
|  |  | $T_{k14}^{pa}$ | 2 | 3 | 8 | 6 | 19 |
|  |  | $M_{k14}^{pa}$ | 5.69 | 7.53 | 20.05 | 15.28 | 48.55 |
|  | $G_{2}$ | $T_{k24}^{wh}$ | 0 | 0 | 0 | 0 | 0 |
|  |  | $M_{k24}^{wh}$ | 0.00 | 0.00 | 0.00 | 0.00 | 0.00 |
|  |  | $T_{k24}^{pa}$ | 0 | 0 | 0 | 0 | 0 |
|  |  | $M_{k24}^{pa}$ | 0.00 | 0.00 | 0.00 | 0.00 | 0.00 |
|  | $G_{3}$ | $T_{k34}^{wh}$ | 0 | 0 | 0 | 0 | 0 |
|  |  | $M_{k34}^{wh}$ | 0.00 | 0.00 | 0.00 | 0.00 | 0.00 |
|  |  | $T_{k34}^{pa}$ | 0 | 0 | 0 | 0 | 0 |
|  |  | $M_{k34}^{pa}$ | 0.00 | 0.00 | 0.00 | 0.00 | 0.00 |
